# Power and pitfalls of computational methods for inferring clone phylogenies and mutation orders from bulk sequencing data

**DOI:** 10.1038/s41598-020-59006-2

**Published:** 2020-02-26

**Authors:** Sayaka Miura, Tracy Vu, Jiamin Deng, Tiffany Buturla, Olumide Oladeinde, Jiyeong Choi, Sudhir Kumar

**Affiliations:** 1 Institute for Genomics and Evolutionary Medicine, Temple University, Philadelphia, PA 19122 USA; 2 Department of Biology, Temple University, Philadelphia, PA 19122 USA; 3 Center for Excellence in Genome Medicine and Research, King Abdulaziz University, Jeddah, Saudi Arabia

**Keywords:** Cancer genomics, Tumour heterogeneity

## Abstract

Tumors harbor extensive genetic heterogeneity in the form of distinct clone genotypes that arise over time and across different tissues and regions in cancer. Many computational methods produce clone phylogenies from population bulk sequencing data collected from multiple tumor samples from a patient. These clone phylogenies are used to infer mutation order and clone origins during tumor progression, rendering the selection of the appropriate clonal deconvolution method critical. Surprisingly, absolute and relative accuracies of these methods in correctly inferring clone phylogenies are yet to consistently assessed. Therefore, we evaluated the performance of seven computational methods. The accuracy of the reconstructed mutation order and inferred clone groupings varied extensively among methods. All the tested methods showed limited ability to identify ancestral clone sequences present in tumor samples correctly. The presence of copy number alterations, the occurrence of multiple seeding events among tumor sites during metastatic tumor evolution, and extensive intermixture of cancer cells among tumors hindered the detection of clones and the inference of clone phylogenies for all methods tested. Overall, CloneFinder, MACHINA, and LICHeE showed the highest overall accuracy, but none of the methods performed well for all simulated datasets. So, we present guidelines for selecting methods for data analysis.

## Introduction

**Figure 1:**
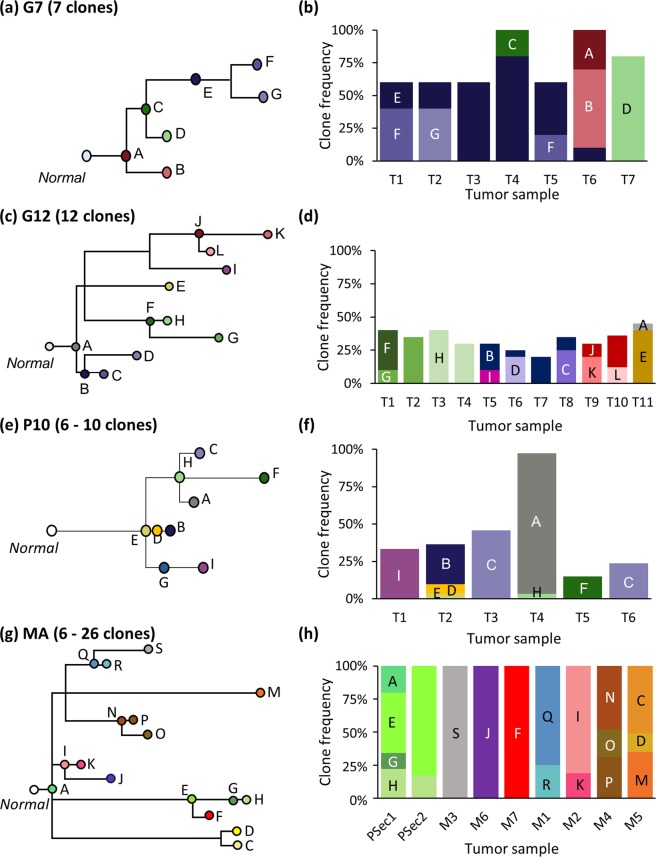
Simulated clone phylogenies and tumor composition. (**a**,**b**) Phylogeny and frequencies of seven clones in seven tumor samples (T1-T7) derived from EV005 tree (G7 datasets)^43^. (**c**,**d**) Phylogeny and clone frequencies of twelve clones and eleven tumor samples (T1-T11) derived from RK26 tree (G12 datasets)^43^. (**e**,**f**) One of thirty phylogenies and its tumor composition from P10 datasets^35^. (**g**,**h**) One example of MA datasets (out of the 60) with primary (PSec. 1 and PSec. 2) and metastatic tumors (M1–M5)^13^. Note that tumor purities are 100% for all the samples.

Somatic mutations play a crucial role in cancer progression^1–3^. Early models proposed that clones with driver mutations sweep through the population, which is the linear progression model of clone evolution^4^. Now, it is clear that tumors are not monoclonal and that the clonal evolution generally follows a branching model (i.e., incomplete clonal sweep) even within a tumor^4–10^. Similarly, clonal evolution in metastatic tumors follows a branching pattern^11,12^. The evolutionary relationship of clones found in primary and metastatic tumors is represented by patient-specific phylogenies^13–16^ (e.g., Fig. 1g,h). The reconstruction and analysis of such clone phylogenies have become standard practices in cancer genomics^16–26^.

At present, clone phylogenies are most often inferred using bulk sequencing data^16,27–30^. Bulk sequencing of tumor samples is cost-effective and can accurately identify single nucleotide variants (SNVs)^31,32^. The resulting data consists of SNV frequencies of cancer cell populations within each tumor sample^27,33^. Several computational methods have been developed to infer individual clone genotypes from SNV profiles and to predict clone phylogenies^13,34–39^. These clone genotypes and phylogenies are then employed to infer relative ordering of somatic mutations and to build migration maps of metastatic tumors^40,41^.

Computational methods for clone prediction and phylogeny inference are operationally different from each other (Table 1). Cloe is a Bayesian method that employs a phylogenetic latent feature model in which clone genotypes are directly inferred by analyzing similarities of observed SNV frequencies^39^. PhyloWGS, another Bayesian method, clusters together SNVs at similar frequencies and then orders them to infer clone genotypes and phylogeny^37^. Metastatic And Clonal History INtegrative Analysis (MACHINA) method follows a process similar to PhyloWGS but incorporates a multi-objective optimization algorithm to jointly infer clone genotypes and a history of cancer cell migration among tumor sites (seeding events) after clustering observed SNV frequencies^13^. The Lineage Inference for Cancer Heterogeneity and Evolution (LICHeE) approach generates SNV clusters defined by the pattern of presence and absence of SNVs among tumor samples while considering SNV frequencies^34^. CloneFinder is a molecular phylogenetic approach that uses presence and absence of SNVs among tumor samples to reconstruct ancestral clones and decompose hybrid clone genotypes in inferring clone genotypes^35^. Treeomics first computes reliability scores for observed SNV and uses only those with high-reliability scores to construct tumor genotypes^36^. Then, it analyzes conflicting mutation patterns in candidate phylogenies of tumor samples and resolves these evolutionarily incompatible patterns in the process of transforming tumor genotypes (presence/absence of mutations) into clone genotypes^36^. MixedPerfectPhylogeny (MixPhy) analyzes only tumor genotypes, ignoring SNV frequencies by using a heuristic algorithm based on co-comparability graphs^38^. It addresses the minimum conflict-free row split problem, where row is tumor genotypes, and observed tumor genotypes are split into clone genotypes. Ultimately, all of these methods deconvolute individual clones from population bulk sequencing of multiple tumor samples acquired over time and/or different locations in a patient.

**Table 1:** Summary of clone prediction methods.

	CloneFinder	MACHINA	TreeOmics	LICHeE	MixPhy	PhyloWGS	Cloe
***Algorithm***							
SNV frequency analysis	Yes	Yes (clustering)	Yes (filtering SNVs)	Yes (clustering)	No	Yes (clustering)	Yes
Binary SNV* analysis	Yes	No	Yes	Yes (clustering)	Yes	No	No
Analysis of evolutionary relationship of tumor sites	Yes (pattern of binary SNV)	Yes (migration of cells)	Yes (pattern of binary SNV)	Yes (pattern of binary SNV)	No	No	No
***Feature***							
Inclusion of SNVs affected by CNAs	No	No	No	No	No	Yes (CNA loci information required)	Yes
One solution	Yes	No	Yes	Yes	Yes	No	Yes
***Accuracy***							
MLTED**	3.80	3.08	4.22	4.23	5.69	6.36	NA***
TreeVec**	0.14	0.13	0.14	0.16	0.28	0.32	NA***
RF**	0.26	0.25	0.40	0.28	0.55	0.47	NA***
Computation time****	<1 min	2 min****	<1 min	< 1 min	< 1 min	8 hours	8 min****

*Presence or absence of SNV in a tumor sample.

**Average across all datasets (G7, G12, P10, and MA). Smaller values are better.

***Cloe did not converge for MA datasets.

****A G7 dataset was used. Computational time of MACHINA depends on the number of solutions produced. For this dataset, it produced small number of solutions (4 solutions). The computational time of Cloe depends on the number of iterations used. For this dataset, a small number of iterations (10,000) was sufficient for the convergence.

Surprisingly, absolute and relative accuracies of clone phylogenies produced by these computational methods have not been assessed using the same collection of datasets, i.e., their performances are yet to be benchmarked. Such benchmarking is critical because of the biological relevance of the downstream inferences derived by using the results produced by these methods. For example, the accuracies of the order of driver mutations and the interrelationship of clones depend on the performance of current methods in accurately deconvoluting individual clone genotypes and reconstructing evolutionary events^13,34,36^. Accurate clone phylogenies are also critical for inferring migration paths. No previous study has evaluated the relative accuracy of clone phylogenies, because their focus has been on introducing and assessing the strengths of the new clone prediction method proposed^13,34–39^. Besides, the robustness of these computational methods to the complexity of clonal structures and evolutionary histories from different tumor sites is mostly unknown.

Therefore, we evaluated the accuracy of clone phylogenies produced by seven methods to predict clone genotypes from bulk sequencing data. We simulated a large number of bulk sequencing datasets under various tumor evolutionary scenarios. Simulated datasets included small and large numbers of persistent ancestral clones as well as metastatic tumors that arose from polyclonal seeding events. Our assessments are based on simulation studies because correct phylogenies are known and because computer simulations have emerged as a standard approach for evaluating the performance of statistical methods in cancer genomics^34,35,37,42^. We evaluated the quality of inferred clone phylogenies by using four different measures, including measures that score the correctness of the order of mutations and those that score the accuracy of the branching order in the reconstructed clone phylogenies. As a result, we have identified limitations of each method and proposed guidelines for researchers to select the most appropriate methods for their data. We have also developed a pipeline (ClonePhyTester; https://github.com/SayakaMiura/ClonePhyTester) that will be useful to test new clone prediction methods by using the performance metrics and simulated data employed in this study.

## Results

We analyzed 330 simulated datasets of tumor bulk sequencing data in which the number of tumor samples ranged from 6 to 11. Tumors and clone sequences were simulated with distinct models of branching evolution (G7, G12, and P10), migrations (MA), and tumor growth (TG). Model clone phylogenies simulated are shown in Fig. 1 for G7 and G12 datasets, and Fig. 3 in ref. ^35^, Supplementary Figs. S1 and S2 for P10, MA, and TG datasets. These simulated clone phylogenies were modeled after those reported in the empirical data analysis^35,43^ (G7 and G12 datasets) or were randomly generated by simulating the birth and death processes of cell lineages (P10, MA, and TG datasets). More details of these simulated datasets are included in the Methods section. We inferred clone phylogenies for each simulated dataset by using seven different methods (Table 1). We used multiple metrics to assess the accuracy, including measures that score the correctness of the order of mutations and the branching order within the reconstructed clone phylogenies.

### Accuracy of ordering mutations

**Figure 2:**
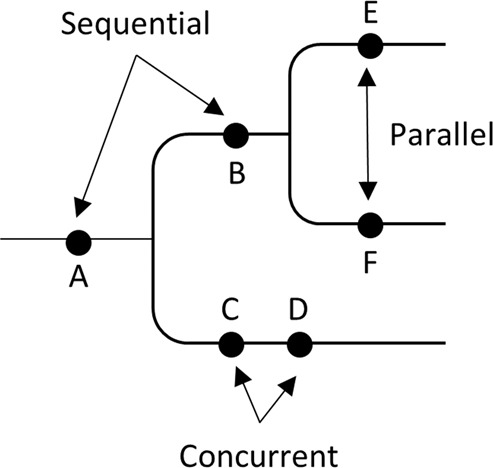
A mutational tree with concurrent (e.g., C and D), sequential (e.g., A and B), and parallel (e.g., E and F) mutations. Dots depict mutations. Order of mutations on a branch (e.g., C and D) cannot be determined based on the clone phylogeny alone.

A clone phylogeny can be viewed as a mutational tree^44^ in which all the mutations are mapped along branches (e.g., Fig. 2). At first, we evaluated the accuracy of the predicted order of mutations by using the MLTED score; a smaller score shows greater similarity between the true and inferred mutational tree (see the Methods section for details). We begin with results for G7 and G12 datasets that were modeled after the predicted evolutionary histories of two patients (EV005 and RK26, respectively) (Fig. 1a–d)^35,43^. Each tumor sample may contain one or a few evolutionarily closely-related clones, assuming a localized genetic heterogeneity^4,6^. That is, the migration of cancer cells to another section of a tumor was assumed to be rare. In total, we obtained 60 simulated datasets (replicates) with 34–89 SNVs per dataset. G7 datasets contained seven tumor samples with seven clones per dataset, while G12 datasets contained eleven samples with twelve clones.

**Figure 3:**
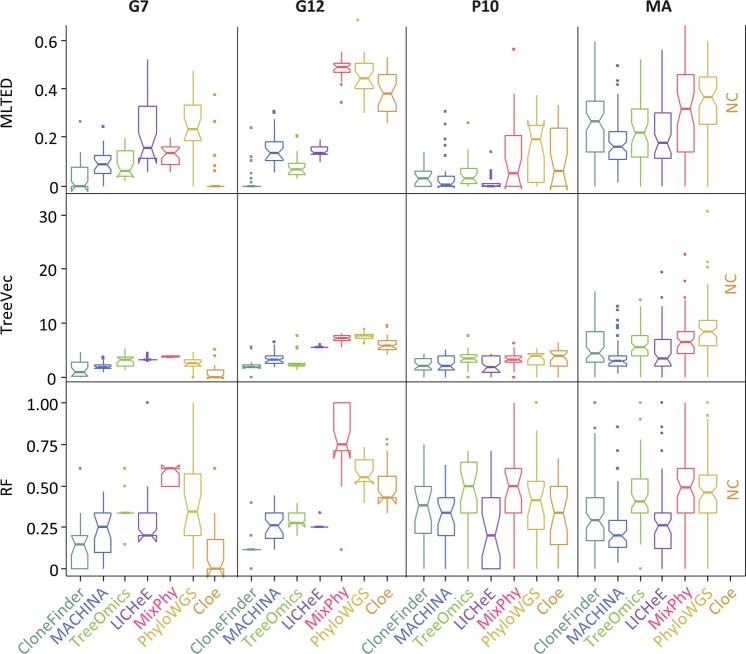
Performance of seven methods measured by MLTED, TreeVec, and RF distances. MLTED scores are for the accuracy of inferred mutation orders, whereas TreeVec and RF measure the accuracy of inferred clone phylogenies (small values indicate higher accuracy). Cloe results were not computable (NC) for MA datasets, because the calculations did not converge.

For the G7 datasets, all seven methods showed relatively small MLTED scores (Fig. 3). Cloe produced much lower MLTED scores than the other techniques for G7 datasets. However, it did not perform well for bigger datasets (G12 datasets). Overall, CloneFinder, MACHINA, Treeomics, and LICHeE outperformed PhyloWGS, MixPhy, and Cloe (Fig. 3). The four best-performing methods consider the evolutionary relationship of tumor samples in making clone predictions (Table 1). These results suggest that the clone prediction methods perform much better when the clone and tumor evolution are coupled and data from many tumor samples are available.

We next examined P10 and MA datasets in which the clonal structures of tumors were more complicated than that in G7 and G12 datasets. In P10 datasets, ancestral clones were present alongside their descendants in tumor samples (Fig. 1e,f). Similar to G7 datasets, MLTED scores of P10 datasets were relatively small for all the methods, but the performance of MixPhy, PhyloWGS, and Cloe was considerably worse than others and showed a large difference among datasets (Fig. 3). Notably, these three methods do not consider evolutionary relationship of tumor samples directly during their inference procedures (Table 1).

The MA datasets were generated by simulating the evolution of primary and metastatic tumors such that more than one founding (seeding) clone migrated from another tumor site(s), which made the clonal structure of metastatic tumors of some datasets more complex (e.g., Fig. 1g,h). For the MA datasets, MLTED scores of all the methods were generally worse (higher) than the other datasets, and MLTED scores varied extensively among the datasets (Fig. 3). Cloe failed to converge even after many days of computations, resulting in a lack of performance values for MA datasets with many clones (see Methods for the detail). MixPhy and PhyloWGS showed slightly worse performance, and MACHINA showed marginally better performance than the other methods. MACHINA is intended for the analysis of primary and metastatic tumors, so it is best suited for MA datasets.

### Accuracy of predicting branching patterns (topology of clone phylogeny)

We next evaluated the accuracy of inferred branching patterns by computing TreeVec and RF distances (see the Methods section for details). These distances measure errors of clone groupings in inferred phylogenies. The results were consistent with those from the analysis of MLTED scores (Fig. 3). For example, in the case of G7 datasets, all the methods generally showed relatively small TreeVec and RF as compared to the other datasets. Indeed, the topologies of reconstructed clone phylogenies were quite similar to the correct phylogeny for these data (Supplementary Fig. S3).

### Accuracy in detecting concurrent, sequential, and parallel mutations

Mutational trees can also be used to test whether a pair of mutations have occurred concurrently, sequentially, or in parallel (Fig. 2). Therefore, we next evaluated error rates of ordering sequential, concurrent, and parallel mutations. We generated all possible pairs of SNVs (mutations) and classified them into concurrent, sequential, and parallel categories. In each group, we computed the proportion of actual mutation pairs that were not present in the inferred tree and the percentage of all incorrect mutation pairs. The average of these two proportions was used to assess the error rate of ordering each type of mutation (see the Methods section for details). For example, the error rate of inferring parallel mutations was computed by using mutations that were classified into parallel mutations.

Overall, error rates of predicted mutation orders (Table 2) showed trends consistent with the results from MLTED, TreeVec, and RF analysis (Fig. 3). However, we found that different types of mutations showed distinct error trends. For G7 and G12 datasets, error rates were similar for all three mutation types, except for MixPhy (Table 2). MixPhy showed an excellent performance in inferring concurrent mutations for G12 datasets, but it performed poorly for sequential and parallel mutations (38% and 15% error rates, respectively). This pattern was caused by the fact that the inferred clone phylogenies were star-like as most of the inferred clones originated from germline cells that did not have any somatic mutations (e.g., Supplementary Fig. S4). Lack of evolutionary structure in the clone phylogeny makes it impossible to detect sequential mutations that spuriously appear as parallel mutations.

**Table 2:** Average error rate of seven methods in inferring order of mutations.

Branch*	Method						
	CloneFinder	MACHINA	TreeOmics	LICHeE	MixPhy	PhyloWGS	Cloe
***G7 dataset***							
Concurrent	2% (4%)	4% (5%)	10% (4%)	20% (11%)	10% (4%)	14% (11%)	0% (0%)
Sequential	4% (5%)	7% (5%)	5% (3%)	17% (10%)	9% (2%)	16% (12%)	2% (6%)
Parallel	3% (5%)	5% (4%)	4% (4%)	19% (17%)	9% (4%)	25% (14%)	2% (6%)
***G12 dataset***							
Concurrent	1% (2%)	5% (3%)	12% (5%)	0% (1%)	0% (1%)	27% (7%)	14% (2%)
Sequential	1% (2%)	7% (4%)	6% (3%)	5% (1%)	38% (2%)	37% (6%)	26% (11%)
Parallel	0% (1%)	4% (2%)	4% (2%)	3% (1%)	15% (2%)	34% (12%)	10% (3%)
***P10 dataset***							
Concurrent	11% (7%)	13% (9%)	13% (9%)	10% (7%)	11% (7%)	13% (7%)	10% (6%)
Sequential	10% (10%)	11% (10%)	11% (11%)	7% (6%)	17% (17%)	19% (13%)	14% (9%)
Parallel	3% (5%)	3% (6%)	1% (4%)	1% (2%)	6% (10%)	11% (13%)	5% (7%)
***MA dataset***							
Concurrent	19% (7%)	13% (8%)	20% (8%)	16% (8%)	19% (7%)	21% (7%)	NA
Sequential	18% (9%)	11% (7%)	18% (8%)	20% (13%)	34% (19%)	27% (12%)	NA
Parallel	5% (5%)	4% (4%)	7% (5%)	5% (5%)	8% (6%)	12% (8%)	NA
Average	6%	7%	9%	10%	15%	21%	NA

Standard deviation is shown in a aprenthesis.

*We generated all possible pairs of mutations (SNVs) and tested if each pair of mutations were correctly ordered along mutational tree. There are three possible orders of mutations, (1) one mutation is placed at ancestral or descendant branch of the other metation (Sequential); (2) mutations are placed at different lineages (Parallel); (3) mutations are placed at a same branch (Concurrent).

We measured the accuracy by computing the error rate, which was the average of the proportion of correct orders not found and that of incorrect orders produced. Smaller values are better. Methods were sorted by the overall average across datasets and branche categories.

For P10 and MA datasets, sequential and concurrent mutations were generally inferred with lower accuracy than the parallel mutations (Table 2). For example, the error rate of inferring parallel mutations was only 4–7% in CloneFinder, MACHINA, Treeomics, and LICHeE analyses for MA datasets, while the error rates for sequential and concurrent mutations were 11–20%. These patterns were observed because many undetected ancestral clones along with a few other recently derived undetected clones were the major errors for P10 and MA datasets, respectively (see later for more detail). These errors would not drastically increase the error rates of parallel mutations but have a strong impact on sequential and concurrent mutations.

### Impact of persisting ancestral clones

**Figure 4:**
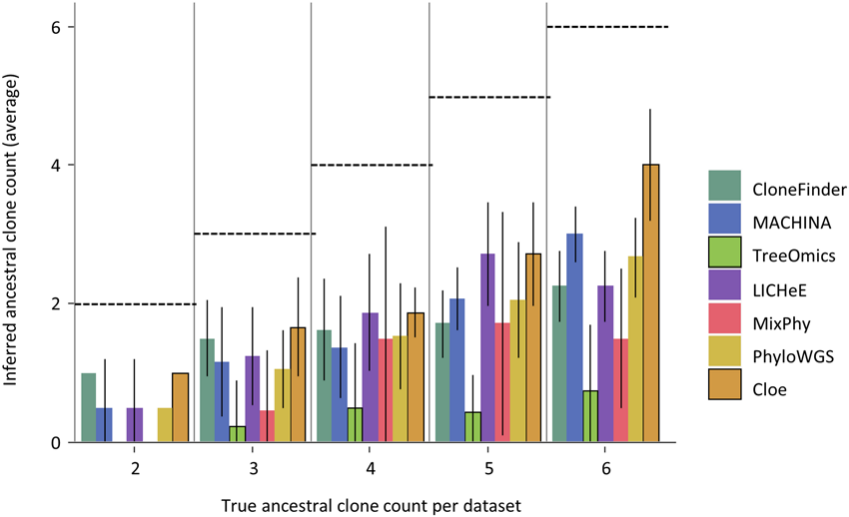
The average number of ancestral clones detected per dataset for the P10 collection. We grouped P10 datasets based on the true number of ancestral clones in a dataset. For each dataset, we counted the number of ancestral clones identified by a clone prediction method. We then computed the average across the dataset. Dashed lines mark correct counts. Error bars represent single standard deviation values.

A unique feature of P10 datasets is the coexistence of ancestral clones with their descendant clones in tumors. We found that fewer than 50% of the ancestral clones were detected by current methods (Fig. 4). Treeomics rarely found any ancestral clone, even in datasets containing as many as six ancestral clones. MixPhy did not do well, either. It is likely because these two methods do not use SNV frequencies in clone predictions, focusing only on the presence/absence of mutations in tumors (Table 1). SNVs found in ancestral clones are expected to show higher SNV frequencies than their descendants, a property that is often used to identify ancestral clones. Therefore, clone prediction methods that do not use observed SNV frequencies (Treeomics and MixPhy) are expected to have difficulty in detecting ancestral clones.

**Figure 5:**
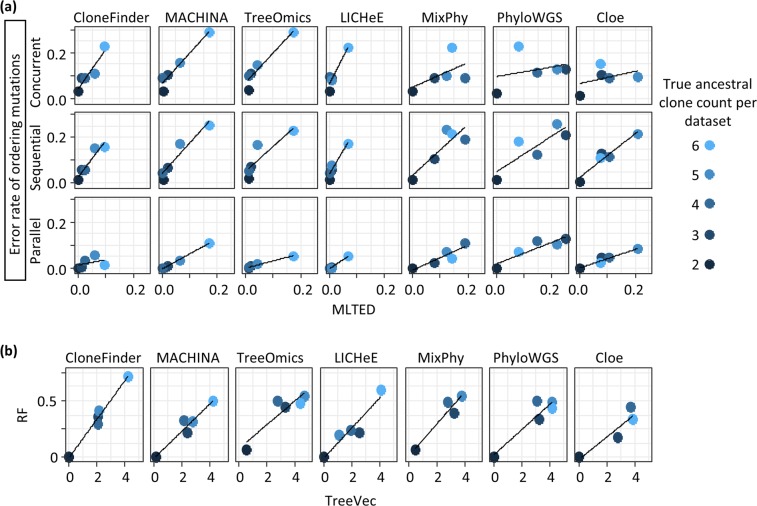
Accuracies of ordering mutations and inferring branching patterns for datasets with different numbers of ancestral clones. P10 datasets were used and were grouped based on the true ancestral clone count in the dataset. Each point shows the average of tree distances across all the datasets in that bin. (**a**) The average error rate of ordering mutations and MLTED scores. (**b**) RF distances and TreeVec scores.

All tested methods performed well in ordering mutations for a dataset that contained only two ancestral clones (Fig. 5a). However, the accuracy of ordering mutations declined when datasets contained tumors with a large number of ancestral clones. In general, the error rate of predicting parallel mutation did not increase significantly with an increasing number of ancestral clones, but the error rates in predicting sequential and concurrent mutations increased significantly (Fig. 5a). As a result, the overall error rates of parallel mutations were lower than the sequential and concurrent mutations for P10 datasets (Table 2). This pattern can be caused by the inability to detect ancestral clones, which will misclassify sequential mutations to be concurrent mutations. Indeed, the missing ancestral clones were the primary difference between the inferred and actual clone phylogenies (e.g., Supplementary Fig. S5). Also, overall TreeVec scores (errors in grouping clones) for P10 datasets were relatively small among the datasets (Fig. 3), indicating that evolutionary relationships of inferred clones were generally accurate.

All tested methods showed relatively small MLTED scores as well as TreeVec and RF distances when a dataset contained only two ancestral clones (Fig. 5). LICHeE, MACHINA, and CloneFinder generally produced smaller TreeVec and RF distances for datasets with larger numbers of ancestral clones (Fig. 5b). Overall, no method produced highly accurate clone phylogenies for datasets containing a large number of ancestral clones due to limited ability to identify coexisting ancestral clones.

### Impact of polyclonal seeding events during metastatic tumor evolution

The analysis of MA datasets was used to assess the impact of polyclonal seeding of metastatic tumors on clone phylogeny and mutation orders. These datasets contained primary tumors with four or six metastatic tumors. Up to four metastatic tumors per dataset evolved with polyclonal seeding events, i.e., these metastatic tumors were founded by more than one seeding clone that came from different clonal lineages (e.g., Fig. 1g,h). In our comparisons, we could not include Cloe because its computation failed to converge for large MA datasets (see the Methods section).

**Figure 6:**
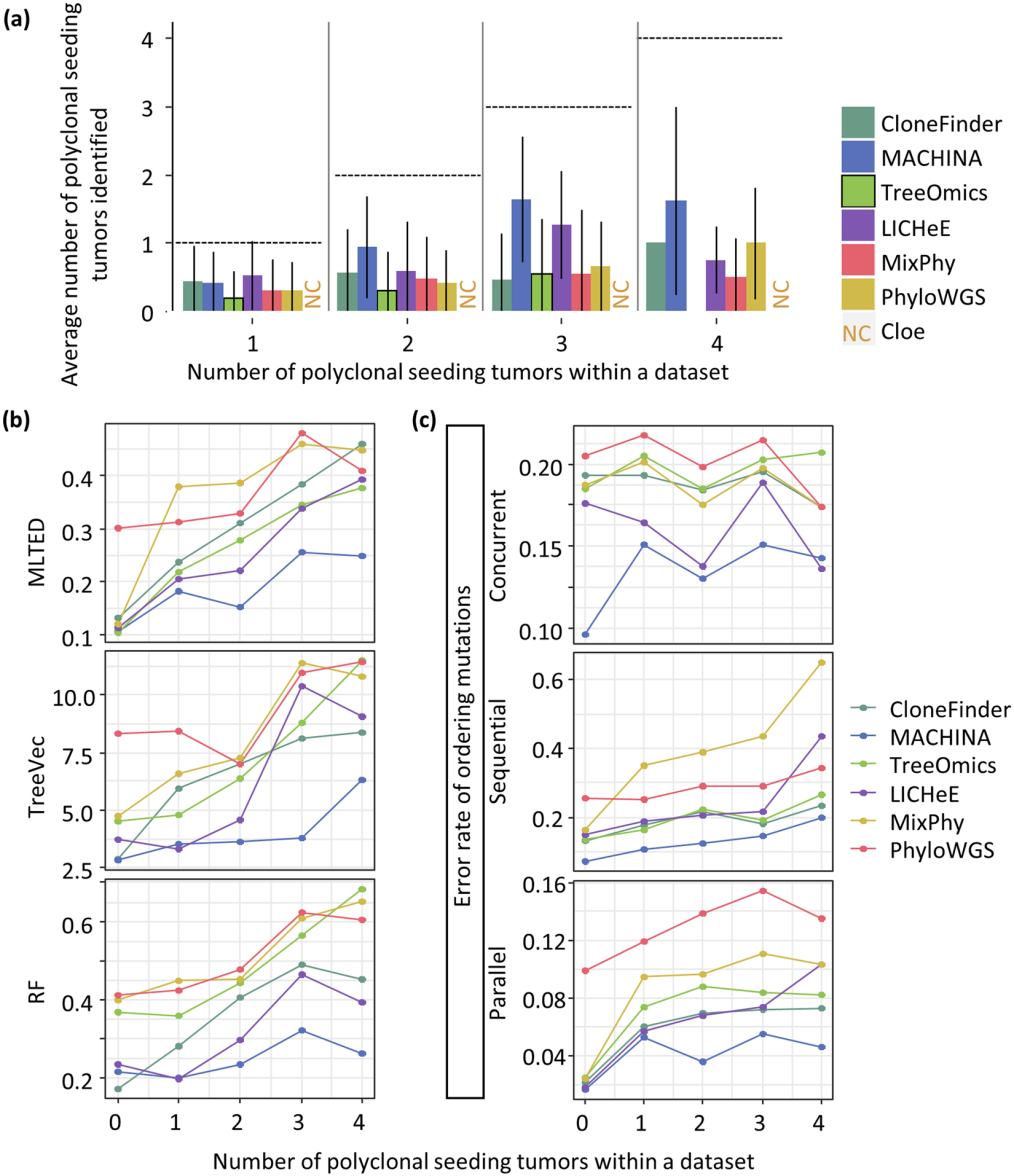
Accuracy of identifying different lineage clones within a tumor for the MA datasets. (**a**) The average count of metastatic tumors with polyclonal seeding events that were predicted. (**b**,**c**) MLTED, TreeVec, RF distances, and error rates of ordering mutations. We excluded Cloe because its computations failed to converge.

None of the tested methods was able to accurately identify a majority of clones within multiple-seeded metastatic tumors (polyclonal metastatic tumors; Fig. 6a). MACHINA was the only method developed to incorporate the metastatic progression model of clone seeding events during its estimation process, and it did outperform other tested methods when datasets contained a large number of multiple-seeding events. However, even for MACHINA, on average, fewer than 50% of the polyclonal metastatic tumors were correctly predicted. Overall, the poor performance of all the methods in inferring clones resulted in higher error rates of ordering mutations and reconstructing clonal branching patterns (Fig. 6b,c).

**Figure 7:**
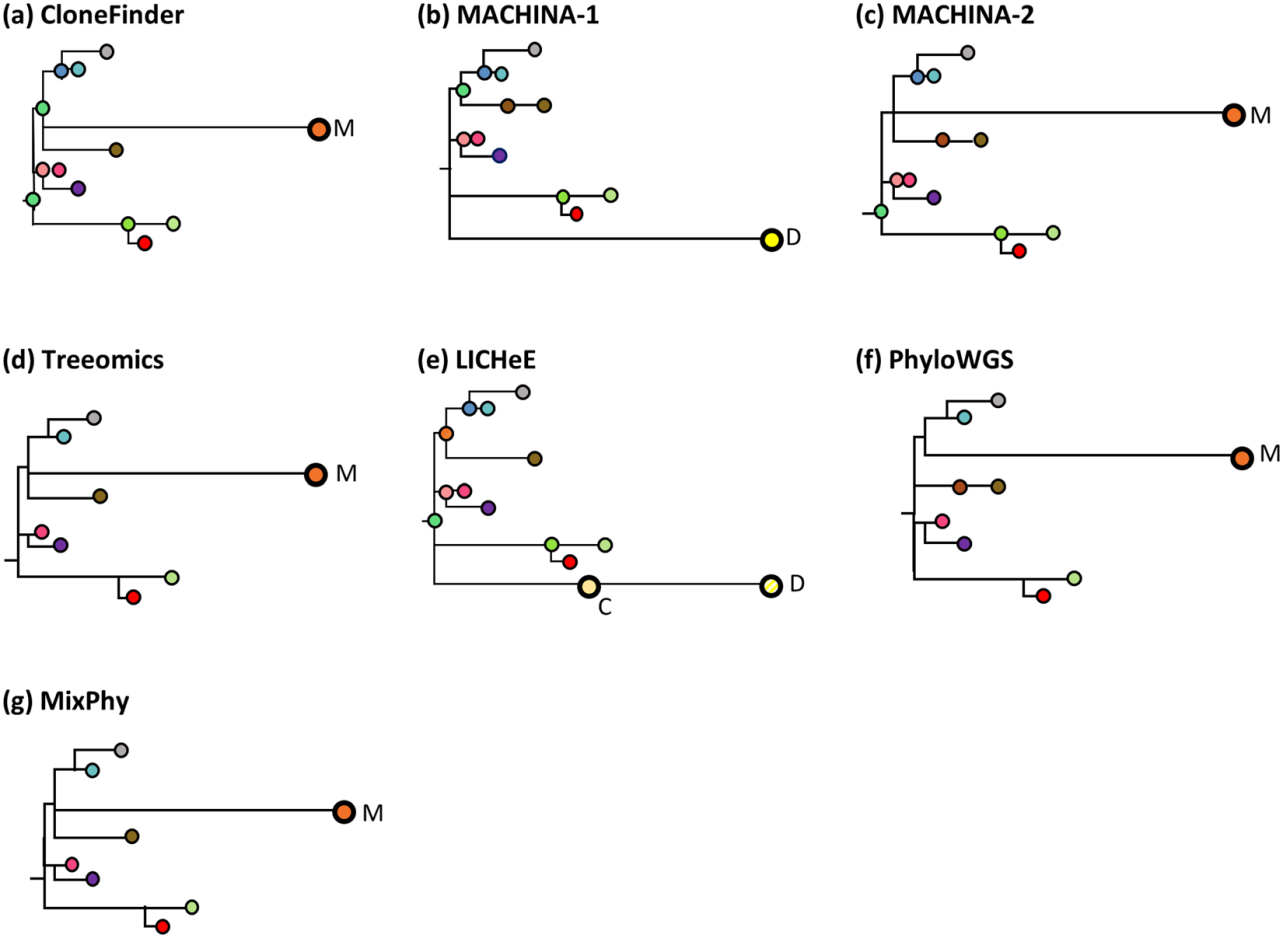
Clone phylogenies inferred by six methods, excluding Cloe for which convergence was a problem, on an MA dataset. True clone phylogeny is given in Fig. 1g. MACHINA produced two solutions (**b**,**c**). Inferred clones are annotated, and colors correspond to clones in Fig. 1g. All the methods produced either clone lineage M or lineage C/D, which were found in the M5 tumor (Fig. 1h). The first solution of MACHINA (**b**) produced clone D, and LICHeE produced clones C and D (**e**). The other methods, CloneFinder (**a**), Treeomics (**d**), PhyloWGS (**f**), and MixPhy (**g**) produced clone M.

Even when an MA dataset contained only one polyclonal seeding event in a metastatic tumor, we observed more errors in phylogenetic predictions that were mainly caused by unsuccessful inference of clones’ presence within that metastatic tumor. For example, Fig. 7 shows inferred clone phylogenies for an example dataset (Fig. 1g,h) in which a metastatic tumor (M5) experienced polyclonal seeding events such that two seeding clones came from two distinct clone lineages (clone lineage C/D, which contained clone C and D, and lineage M with clone M). All the methods, including MACHINA, identified only one out of these two clone lineages (lineages C/D or M).

MACHINA produced two solutions (Fig. 7b,c). The first solution contained only clone C, and the second solution provided only clone M. In these MACHINA phylogenies, each of these clones was connected with an erroneously long branch (Figs. 1g and 7b,c). This is because those correct clones found within the M5 metastatic tumor were combined into one clone genotype in the inferred clone phylogenies. This same type of error was observed in predicted clone phylogenies generated by other methods as well (Fig. 7). Apart from these errors, the predicted clone phylogenies were mostly similar to the true clone phylogeny, and the branching patterns were generally correct (Figs. 1g and 7). For this example MA dataset, MACHINA, CloneFinder, and LICHeE produced more accurate clone phylogenies than other methods. For instance, Treeomics, PhyloWGS, and MixPhy produced phylogenies with many fewer clones, as these methods failed to detect many ancestral and highly-similar clones. We found that these types of errors were more common in datasets with many polyclonal seeding events. For example, when a dataset was composed of four metastatic tumors with polyclonal seeding events, inferred clone phylogenies contained fewer clones than the true phylogeny (Supplementary Fig. S6). All methods tended to predict only one clonal lineage for every polyclonal metastatic tumor in this dataset. Therefore, currently available methods will tend to underestimate the numbers of polyclonal seedings of tumors.

Interestingly, MACHINA produced 870 phylogenetic solutions for this example dataset. We examined the best and worst solutions based on the number of SNV assignment errors per clones. That is, the best solution had the smallest number of SNV assignment errors. We found that the phylogeny of the best solution looked very similar to the true phylogeny because it correctly identified most of the clone lineages (Supplementary Fig. S6). However, the phylogeny of the worst solution contained a large number of errors, like some other methods. At present, in the real data analysis, MACHINA does not provide any way of selecting among the 870 solutions.

Error rates for ordering parallel mutations tended to be lower than that for sequential and concurrent mutations, regardless of the number of tumors with polyclonal seeding events (Fig. 6c). This resulted in lower overall error rates in detecting parallel mutations (Table 2). We can trace these errors to the long branches leading to tip M in Fig. 7, which are caused by the fact that clone M now also contains mutations of two sister clones (C and D in Fig. 1g). Such hybrid errors cause sequential mutation to be underestimated and concurrent mutations to be overestimated. Also, MA datasets tended to contain a large number of clone lineages (Fig. 1g and Supplementary Fig. S1). Consequently, the numbers of parallel mutation pairs in the correct phylogenies were much larger than sequential and concurrent mutations (Supplementary Fig. S7). Thus, missing one or a few clone lineages in inferred clone phylogenies does not significantly affect the error rates for identification of parallel mutations as compared to sequential or concurrent mutations. Overall, current clone prediction methods cannot reliably decompose many clones within metastatic tumors that have experienced polyclonal seeding events.

### Effect of the intermixture of cancer cells in tumor samples

**Figure 8:**
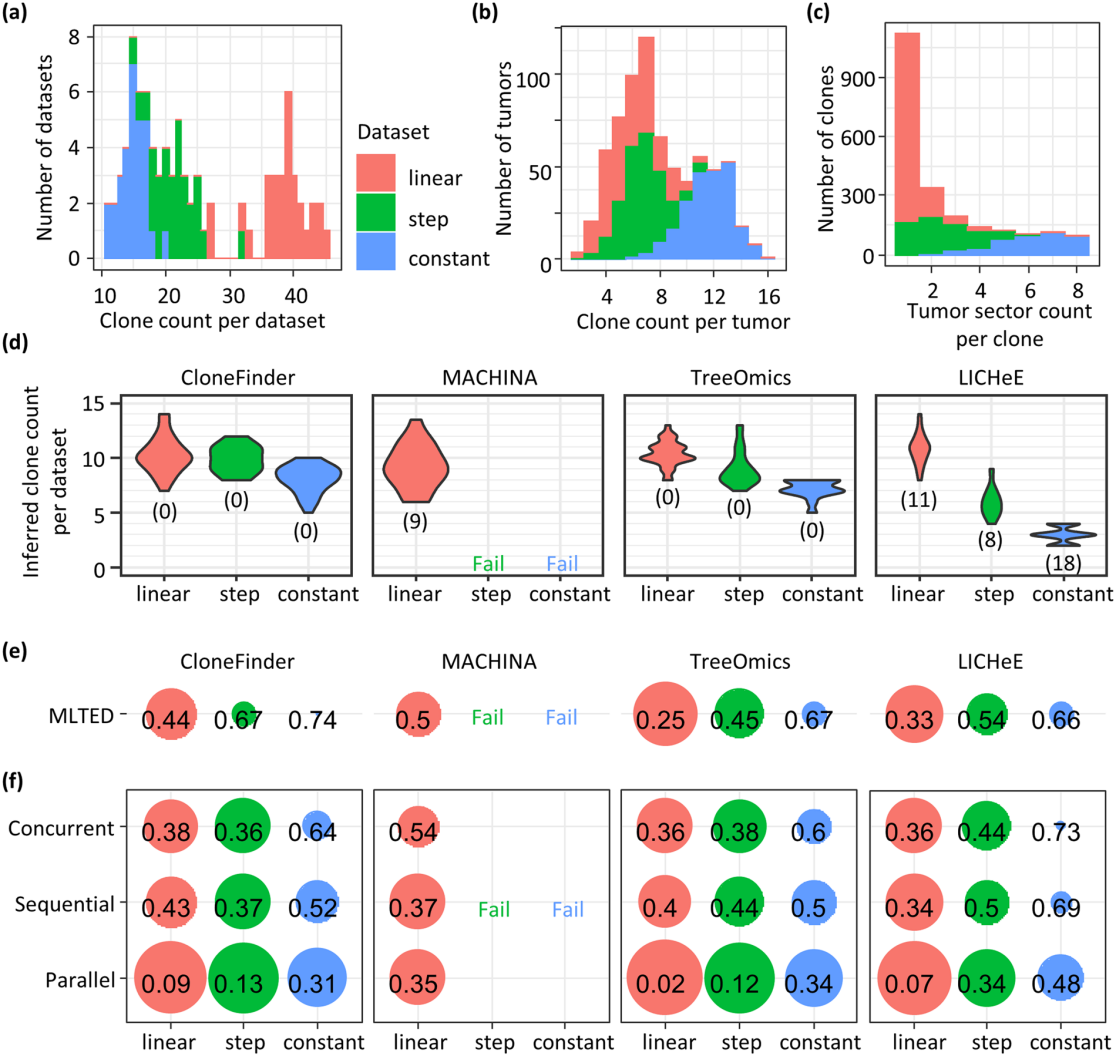
Accuracy of inferred clone phylogenies for TG datasets (linear, step, and constant). (**a**) The total number of clones in a dataset. (**b**) The total number of clones within a tumor sector (sample). (**c**) The number of tumor sectors that had a clone, which showed the degree of clone sharing among tumor samples. (**d**) The total number of inferred clones for a dataset. The number in parenthesis is the number of datasets that a method failed to produce a result. (**e**) The average MLTED score. A smaller value is better. (**f**) Error rates of ordering mutations.

We further tested how the intermixture of cancer cells between tumor sites (or between sections of a tumor) decreases the accuracy of clone phylogeny inferences. We generated 90 TG datasets (Supplementary Fig. S2) by simulating tumor growth with three distinct evolutionary scenarios that resulted in different degrees of intermixture (see Methods for the detail). We further classified these TG datasets into TG-constant, TG-step, and TG-linear datasets, in which the TG-constant datasets exhibited the highest degree of intermixture of cancer cells among the TG datasets because the cell division rate was set to be the same for all the cancer cells. In TG-linear datasets, cancer cell populations expanded linearly resulting in the lowest degree of intermixture, because new cancer cells are not produced where the other cancer cells are already present. The model used for generating the TG-step datasets produced intermediate degree of intermixture of cancer cells, as the rate of cell division was constant while daughter cells were produced only when a cell was not all surrounded by the other cells. These strategies resulted in clones being widely distributed within a tumor for a TG-constant dataset, i.e., the same clones were found within many different tumor sectors (samples), or many clones were found only within single sectors of a tumor for the TG-linear dataset (Fig. 8a–c).

LICHeE computation failed for >40% of the TG datasets, and it severely underestimated the number of clones (2–4 instead of 10 clones) for TG-constant datasets (Fig. 8d). It performed the best for TG-linear datasets, which is reasonable as there was less mixing of clones across samples. The use of MACHINA generally produced >100 solutions (even thousands of solutions for a few datasets; Supplementary Fig. S8), with no way of selecting among them for all the TG-constant and TG-step datasets. Since analysis of hundreds of equally plausible solutions in actual empirical data analysis is not practical, we considered that MACHINA failed on these datasets (see Methods). However, it performed well for TG-linear dataset, just as LICHeE did.

Interestingly, CloneFinder produced many more clones than other methods for TG datasets (Fig. 8d), even though all the clone prediction methods underestimated clone counts (Fig. 8d compared with Fig. 8a). Consequently, inferred clone phylogenies were much smaller than the true phylogenies, which was consistent with the results of MA datasets. TG-constant were the most difficult to analyze (highest MLTED scores; Fig. 8e), reflecting that degree of the intermixture of cancer cells among samples being a significant determinant of the success (Fig. 8c). Also, mutation orders were more accurately inferred for TG-linear datasets than TG-step and TG-constant datasets (Fig. 8f). Therefore, these results confirmed that the clone phylogeny inference is challenging when intermixture of cancer cells is extensive.

### Impact of low sequencing depth

We also tested if the clone prediction methods were robust to sequencing depth. We examined the change in their performance when the sequencing depth was 50x, as compared to 200x initially simulated in the MA datasets. We selected MA datasets because a growing number of investigators are performing tumor bulk sequencing to infer clone phylogenies of primary and metastatic tumors in patients^14,22,45,46^.

**Figure 9:**
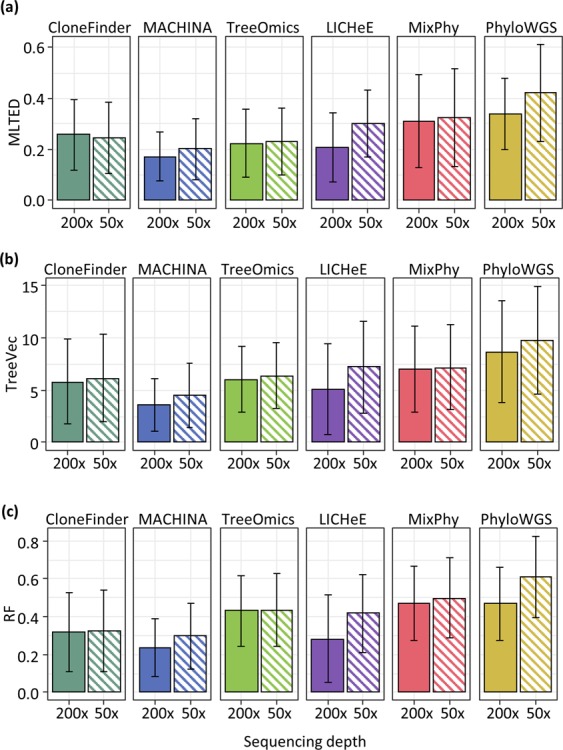
Comparison of the performances of clone prediction methods with low sequencing depth (50x). Average MLTED (**a**), TreeVec (**b**), and RF (**c**) scores were compared between datasets with 200x and 50x coverage. MA datasets were used for those with 200x coverage. We generated 50x datasets by using true clone genotypes and clone frequencies of MA datasets. Error bars indicate a single standard deviation.

We found CloneFinder, Treeomics, and MixPhy to work well for 50x datasets, and MLTED, RF, and TreeVec scores generally did not change significantly (Fig. 9). However, MACHINA, LICHeE, and PhyloWGS were affected considerably by sequencing depth (Fig. 9). For example, MLTED score became worse by 0.03, 0.09, and 0.08 on average for these methods (*p *≪ 0.01), while the other methods showed <0.01 change in MLTED scores. Computational methods that were not affected by low sequencing depth did not use observed SNV frequencies to cluster SNVs in their clone prediction, unlike those that suffered a decline (Table 1). Observed SNV frequencies are less reliable when the sequencing depth is low^34,36^, which adversely impacts the inference of clusters of SNVs that are used by MACHINA, LICHeE, and PhyloWGS as primary building blocks in inferring clone phylogenies.

### Impact of CNAs and LOH

The focus of our investigation is primarily on the use of copy-neutral SNVs, because most of the methods cannot handle copy number alterations (CNAs; Table 1). So, except for Cloe^39^, all other methods require prefiltering steps to exclude SNVs affected by CNAs (PhyloWGS requires that SNVs affected by CNAs be specified; Table 1). We conducted a preliminary investigation of how the inclusion of SNVs affected by CNAs and the loss of heterozygosity (LOH) impacted various methods, especially because the identification of CNAs is still a challenge for bulk sequencing data^47–49^.

**Figure 10:**
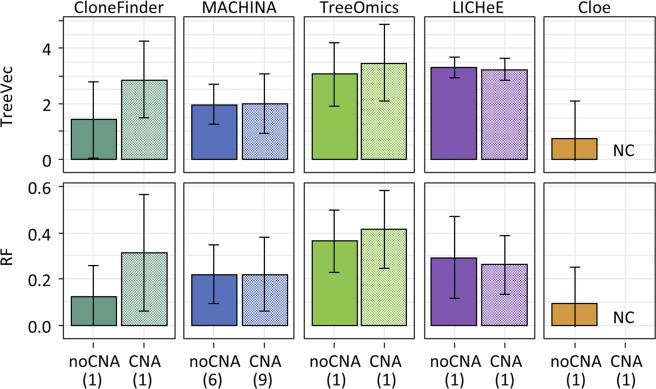
Comparison of the performances of clone prediction methods with datasets that contained CNAs. Average TreeVec and RF scores were compared between datasets without CNAs (noCNA) and those with CNAs. G7 datasets were used for those without CNAs. We generated G7-CNA datasets by introducing CNAs. Error bars indicate one standard deviation. The number in parenthesis is the average number of solutions per dataset.

We introduced CNAs and LOH in G7 datasets (G7-CNA), which was done because Cloe could be used for these datasets when copy-neutral SNVs were used for G7 (see Methods for the detail). On average, seven SNVs per clone were affected by a CNA and/or LOH. Unfortunately, Cloe did not converge for G7-CNA datasets even after many days of calculations (see also Methods). As expected, the accuracy of all the methods was relatively low for these data, with previously best-performing methods experienced large declines for G7-CNA datasets (Fig. 10). MACHINA tended to produce a larger number of solutions for G7-CNA datasets than for G7 datasets (9 and 6 solutions per dataset on the average). Overall, the presence of CNA and LOH has a substantial adverse impact on well-performing methods. More extensive benchmarking is needed in the future to better understand accuracy trends for available methods.

### Ensemble approach

**Figure 11:**
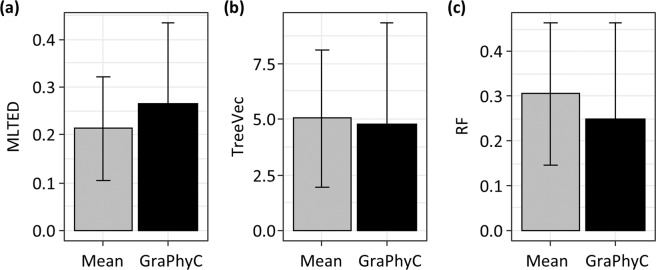
Accuracy of consensus trees produced by GraPhyC, for which we used trees produced by CloneFinder, MACHINA, TreeOmics, and LICHeE. Average MLTED (**a**), TreeVec (**b**), and RF (**c**) scores of GraphyC across datasets are shown together with the average scores from the use of CloneFinder, MACHINA, TreeOmics, and LICHeE separately (“Mean”). MA datasets were used. Error bars indicate single standard deviations. GraPhyC results are from applying the “path” option, but the other three options (“ancestor-descendant”, “clonal”, and “parent-child”) produced very similar results.

We also evaluated the performance of GraPhyC, a new method to build a consensus clone phylogeny from multiple clone phylogenies^50^, to test if an ensemble approach can improve the performance. GraPhyC analyzes input mutation trees and produces a consensus tree that shows the highest similarities to all the input trees^50^. In these analyses, we used MA datasets because all the methods showed the most error for these datasets. We selected CloneFinder, MACHINA, Treeomics, and LICHeE as input to GraPhyC because these four methods produced the lowest mutation distances (MLTED scores; Fig. 3). We found that the MLTED scores of GraPhyC consensus trees were slightly worse than the average scores of the four methods, but the consensus trees showed slightly better TreeVec and RF scores (Fig. 11). The lack of extensive improvement is partly attributable to the fact that a significant source of error in all the methods is their inability to detect correct clones. Because GraPhyC does not identify clones but instead builds consensus trees, it is expected only to improve clone groupings.

### Empirical data analysis

**Figure 12:**
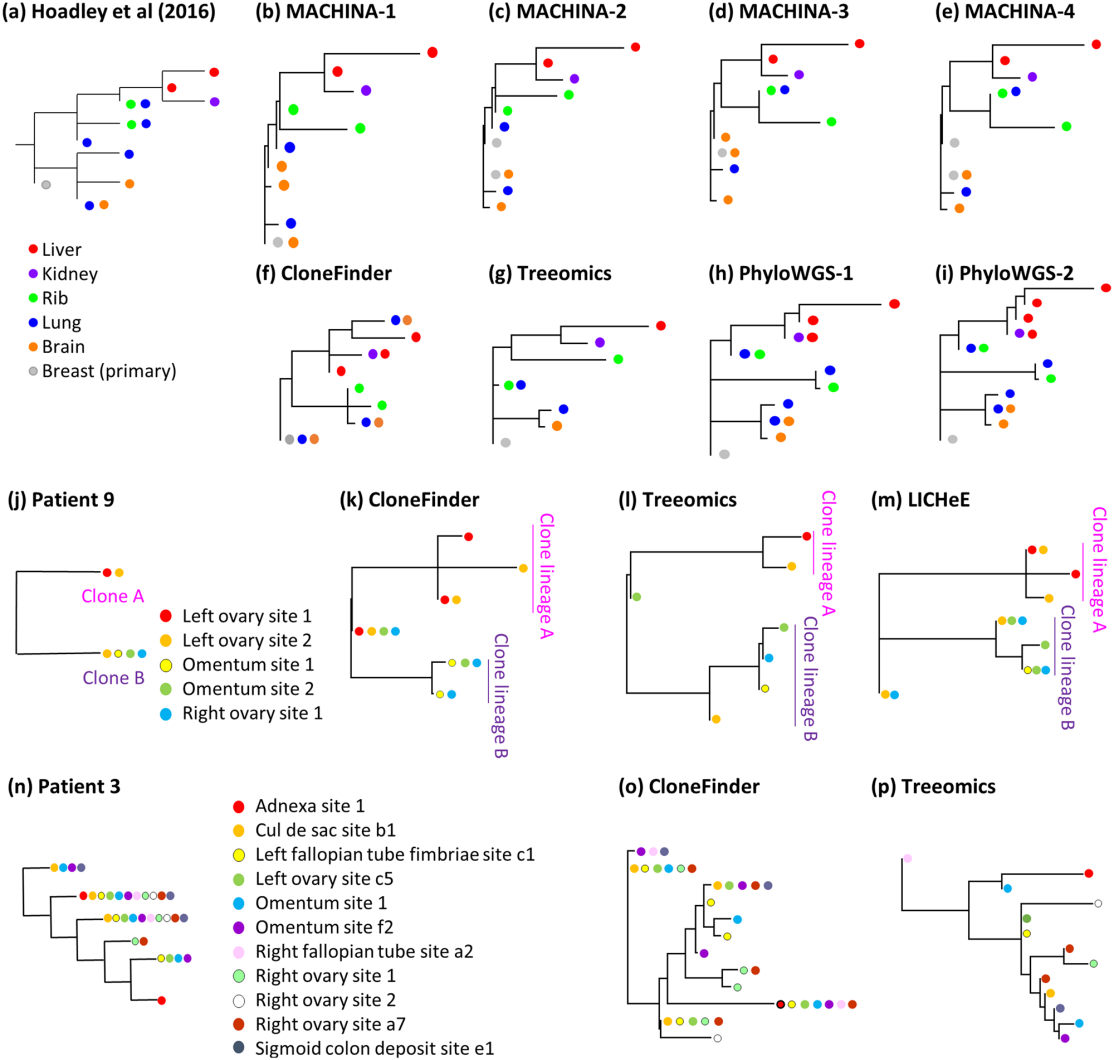
Empirical data analysis for the Hoadley *et al*. (2016) dataset (A7) (**a**–**i**), Patient 9 (**j**–**m**), and Patient 3 datasets (**n**–**p**). The color of clones in the phylogeny corresponds to the location of clones’ samples. (**a**,**j**,**n**) Clone phylogenies reported by Hoadley *et al*. (2016) (**a**) and those by McPherson *et al*. (2016) (**j**,**n**). (**b**–**i**) Inferred clone phylogenies by using (**b**–**e**) MACHINA, (**f**,**k**,**o**) CloneFInder, (**g**,**l**,**o**) Treeomics, (**h**,**i**) PhyloWGS, and (**m**) LICHeE. MACHINA and PhyloWGS produced more than one phylogeny for the A7 dataset.

The application of these clone prediction methods to an empirical dataset (A7 dataset from a previous study^30^) showed results consistent with our analyses of simulated data. The original study reported that metastatic rib and lung tumors harbored clones from different clonal lineages (Fig. 12a). The lung tumor contained three different clone lineages, indicating a complicated history of metastatic tumor evolution. Different methods predicted clone phylogenies that showed limited similarity to the clone phylogeny reported in the original study (Fig. 12b–i). MACHINA produced four similar solutions (Fig. 12b–e). However, only the predicted evolutionary relationship of clones from liver and kidney tumors agreed with those reported in the original study^30^. The predicted clone sharing between lung and brain tumors reported by CloneFinder agreed with the initial research, but the clone phylogeny differed dramatically (Fig. 12f). Treeomics correctly predicted the evolutionary relationship of clones from the liver, kidney, and rib tumors, but did not predict most of the ancestral clones (Fig. 12g), a failing that we also observed in our simulation results. PhyloWGS produced two distinct but highly similar clone phylogenies (Fig. 12h,i) that indicated the presence of three clonal lineages, instead of the two lineages reported in the original study. LICHeE analyses did not produce a solution. MixPhy produced >400 clones for this dataset. Cloe failed to provide a solution due to the lack of computational convergence. Therefore, we anticipate that the application of different computational methods in actual empirical data analysis will result in widely varying inferences, making it challenging to reach reliable biological conclusions when the tumor evolution is highly complex.

We also tested the impact of CNAs by using two empirical datasets (patient 3 and 9 datasets from high-grade serous ovarian cancer^51^). In these datasets, a large number of SNVs are affected by CNAs, because the ploidy of cells in a sample was reported to be ~3.5^51^. Application of MACHINA to these datasets was unsuccessful, as the computation did not finish even after days of computation. For other methods, the inferred clone phylogenies showed many similarities to that reported in the original study, when the structure of clone phylogeny was simple, i.e., patient 9 dataset (Fig. 12j–m). For this dataset, only two clones were reported in the original study^51^, and each tumor site contained only one of these two clones, except for the left ovarian site 2, which had both of the clones (Fig. 12j). CloneFinder, Treeomics, and LICHeE predicted these two clones, which were confirmed by single-cell sequencing in the original study. Computational methods also predicted the presence of the root clone in the patient sample, as well as other clones that were closely related to the two clones reported in the original study (clones in clone lineage A and B in Fig. 12k–m). The original authors only reported two clones, and their single-cell sequencing was focused on validating those two clones only. In the future, it will be interesting to learn if the new clones predicted by computational methods are found in their samples. For the Patient 3 dataset, the original study reported that all of tumor samples contained at least two clones, and many clones were predicted to be found more than one tumor site (Fig. 12n). Thus, the reported clonal structure was more complex than that for patient 9 (Fig. 12j). LICHeE failed to produce any results for this dataset, and CloneFinder and Treeomics produced phylogenies that looked very different from those reported in the original study (Fig. 12o,p). Therefore, CloneFinder and Treeomics performed poorly on datasets with CNAs, as we found in the computer simulations. Therefore, the utility of current methods for datasets with ample CNAs is limited.

### Computation time

Lastly, we examined the computation time. Methods that produce a single solution per dataset are generally faster (CloneFinder, TreeOmics, LICHeE, and MixPhy) than those that produce multiple solutions (MACHINA and PhyloWGS) (Table 1). For example, these fast methods required <1 minute for a G7 dataset. The only exception among the methods that produce a single solution for a dataset was Cloe, which required a longer computational time than MACHINA for a G7 dataset. It is reasonable because Cloe is a Bayesian method, in which a large number of iterations are necessary for each data analysis. PhyloWGS is also a Bayesian method, and it was slower than MACHINA for a G7 dataset (Table 1). In the case of MACHINA, the number of solutions produced per dataset extensively varied among datasets, some of which produced ≫100 solutions. Since MACHINA analyzes the history of migration events for each solution, the computational time tended to increase as the increasing number of solutions, e.g., TG datasets (see Methods for the detail).

## Discussion

Predictions of accurate clone phylogenies are essential to infer the order of driver mutation occurrences and the evolutionary relationship of clones. We tested the accuracy of published methods in reconstructing clone phylogenies as a first step in identifying the patterns of errors in clone phylogeny inference. We observed that clone phylogenies produced by some methods (CloneFinder, MACHINA, Treeomics, and LICHeE) were often more accurate than the other methods, i.e., lower MLTED, TreeVec, RF, and the error rate of ordering mutations. These results were consistent with the accuracy of inferred clone genotypes, i.e., the number of SNV assignment errors per clone (Supplementary Table S1)^35^. Based on the results of our simulation studies, we propose a few useful guidelines for applying computational methods in practical data analysis of SNVs obtained from bulk sequencing data of multiple tumor samples.

To begin with, we suggest the use of CloneFinder, MACHINA, Treeomics, or LICHeE, because they often performed the best in ordering mutations and inferring phylogenies (Fig. 3 and Tables 1 and 2). All of these methods benefit from the use of the intrinsic evolutionary relationship of tumor clones (Table 1). The evolutionary information provides resolution beyond inferences primarily based on the dissimilarities of observed SNV frequencies because low read depths will cause SNV frequencies to be less accurate and clone predictions based on only the similarities of observed SNV frequencies will become error-prone. Also, the intrinsic phylogenetic information among tumor samples is likely to be higher for datasets with larger number of samples, so these four perform even better (e.g., G12 datasets). Importantly, datasets with a very small number of samples will underestimate the genetic heterogeneity of a tumor site, and therefore, the use of a large number of samples per patient is often recommended^6,52^.

When tumor sectors or sites are anticipated to exchange cancer cells frequently (e.g., frequent polyclonal seeding events for metastatic tumors), most of the clones will be shared among samples, e.g., serially sampled chronic lymphocytic leukemia^53^. In this case, one may choose to use MACHINA and LICHeE, because these methods are marginally preferable over CloneFinder and TreeOmics (MA datasets). In particular, techniques that strongly depend on the observed patterns of presence/absence of mutations among tumor samples (e.g., Treeomics and CloneFinder) will have difficulty detecting clones correctly^35^, resulting in less accurate clone phylogenies. However, when intermixture is substantial, none of the clone prediction methods are likely to predict clone phylogenies accurately (TG datasets). A previous study also reported that the inferred clone genotypes are inaccurate on such datasets^35^. Since MACHINA tended to produce a large number of solutions and/or required a very long computational time for datasets with an extensive intermixture of clones among samples (TG datasets), this condition can be used to identify datasets, in which inferred clone phylogenies are potentially erroneous.

By contrast, when the sequencing depth is low (e.g., 50x), we recommend methods that do not primarily rely on observed SNV frequencies in clone predictions, such as CloneFinder and Treeomics. However, most of the methods are known not to be robust to the presence of incorrect SNV assignments, so one should proceed with extreme caution when analyzing datasets with high rates of sequence error. We suggest excluding, or correct potentially erroneous mutation detections, by using computational tools such as those implemented in Treeomics^36^. Especially, LICHeE may fail to produce any inferences on such datasets, or the accuracy may become much lower than other methods (e.g., Treeomics)^36^. In fact, LICHeE failed to produce any results for our example empirical dataset^30^. Also, SNVs that are affected by copy number alterations (CNAs) should be excluded, as most of the clone prediction methods require copy-number-neutral SNVs.

Also, we suggest using multiple methods to infer clone phylogenies and examining the consistency among the results. We observed that the best performing methods produced similar results when the inferred clone phylogenies were accurate. When using Treeomics, it is crucial to be aware that the inferred clone phylogenies will not contain most of the ancestral clones. Also, potential errors on clonal lineage deconvolution can be detected when MACHINA produces at least two different clone phylogenies (e.g., Fig. 7) or when MACHINA provides hundreds of solutions. In general, the inconsistency of inferred clone phylogenies is an indication of the presence of mixing of different lineage clones in tumors. Currently, no methods produce accurate clone phylogenies from such data. Thus, consistency among inferred phylogenies may be useful to validate inferences. Consensus phylogenies by using GraPhyC^50^ may then be helpful to find clone groupings that are detected by multiple methods or to computationally summarize various solutions produced by a clone prediction method.

The above initial guidelines will undoubtedly evolve as new methods are developed to detect clones and phylogenies and new metrics are designed to assess performance. To facilitate comparative benchmarking of these new methods with those tested here using the simulated data employed in our investigations, we have made available a pipeline (ClonePhyTester; https://github.com/SayakaMiura/ClonePhyTester). ClonePhyTester uses the clone sequences output by the new method and calculates all the performance metrics as well as graphical visualizations and summaries that will directly compare any new techniques with the seven methods we tested. For more advanced analyses, ClonePhyTester can be modified and expanded because it is programmed in widely used Python language.

## Conclusions

Analyses of correct clone phylogenies are critical to a better understanding of tumor evolution and the origin and extent of genetic heterogeneity in tumors. We can accurately infer clone phylogenies only when tumor evolution generally tracks clonal evolution, a relationship that is disrupted when tumors exchange clones. This disruption, along with the persistence of many ancestral clones that persist alongside their descendants within tumors, makes it challenging to detect clones and reconstruct evolutionary history of clones and ordering of mutations. The use of multiple methods and consensus inferences have the potential to validate predictions of specific methods and to detect problematic results. However, there is a strong need for more advanced techniques that can perform well for datasets that show intermixing of tumor samples.

## Methods

### Generation of bulk sequencing data

We analyzed 330 simulated datasets, and all of these datasets were available from https://github.com/SayakaMiura/ClonePhyTester. Each dataset contained information on mutant and wild-type read counts (with read counting errors).

#### G7 and G12 datasets

These datasets were obtained from ref. ^35^ and contained seven and twelve clones, respectively, modeled after the predicted evolutionary histories of two patients (EV005 and RK26^43^, respectively) (Fig. 1a–d)^35^, i.e., we used the same topologies of clone phylogenies that were reported in the original study. Each tumor sample may contain one or a few evolutionarily closely-related clones, assuming a localized genetic heterogeneity due to branching evolution^4,6^. Thus, the migration of cancer cells to another section of a tumor was assumed to be rare in these datasets. In total, we obtained 60 simulated datasets (replicates) with 34–89 SNVs per dataset.

#### P10 datasets

P10 datasets were also obtained from ref. ^35^. In these datasets, various numbers of clones persisted within a sector (sample) of a tumor after the origin of descendant clones. Ten random clone phylogenies were simulated, with the consideration of the birth and death process of cell lineages, in which a random number of mutations were assigned at each branch of a phylogeny^54^. Every tumor sample was populated with one tip clone and its ancestral clones^35^, following the “localized sampling process” in ref. ^34^ (Fig. 1e,f). Each of P10 datasets contained 2–6 ancestral clones (30 datasets). A selection of simulated clone phylogenies is shown in Fig. 3 of Miura *et al*.^35^.

#### MA datasets

These datasets were obtained from the MACHINA website (https://github.com/raphael-group/machina) and were generated by modeling the evolution of primary and metastatic tumors (four or seven metastatic tumors per dataset)^13^. Metastatic tumors were founded by cancer cells (seeding clones) that migrated from another tumor site (primary or another metastatic tumor). Under a simple metastatic tumor evolution scenario, each metastatic tumor received a single founder (seeding) clone from another tumor site, and a metastatic tumor contained only clones that evolved from a single seeding clone. Clonal structures of metastatic tumors became more complicated when a metastatic tumor was seeded by more than one clone (polyclonal seeding events). In MA datasets, a metastatic tumor received a maximum of two seeding clones, and any dataset may contain more than one metastatic tumor with polyclonal seeding events. Thus, the observed genotypes of these metastatic tumors represented two convoluted clone lineages, and clone prediction methods were required to correctly identify such tumors and decompose them into two distinct clone lineages (e.g., Fig. 1g,h). Each MA dataset contained up to four metastatic tumors with polyclonal seeding events. Each clone phylogeny was unique (60 MA datasets). All the clone phylogenies are shown in Supplementary Fig. S1.

#### TG datasets

We simulated three-dimensional tumor growth by using tumopp software^55^. We used a hexagonal lattice to arrange the location of cancer cells in space. The shape parameter (*k*) for the gamma distribution of waiting time for cell divisions was set to 10, and we used the default potential cell division rate = 1. Three different models were used for cell division, (a) linear-function model, in which the birth rate of a new cancer cell was proportional to the emptiness of its surrounding space, (b) step-function model, in which cell division occurred only when a cell was not surrounded by the other cells, and (c) constant-rate model, in which the birth rate was constant regardless the presence of other cells at the surrounding space. Based on these models, we classified TG datasets into linear, step, and constant datasets, respectively. Each simulation was terminated when the number of extant cancer cells became 10,000. We used the default values for the other parameter settings, i.e., all cells were assumed to be stem cells, push model was randomly assigned, the cell death rate and migration rate were zero, and driver mutations were not introduced. We then randomly selected eight sections (sectors) of a tumor that were uniformly located. From each sector, 100 cancer cells were sampled. All cells in a tumor were assumed to be cancerous, as tumopp did not simulate normal cells. Please note that an actual tumor and a sample should contain much larger number of cancer cells than we generated in this simulation.

To generate cancer cell sequences (genotypes), we introduced mutations during cell divisions such that the number of mutations per cell division was sampled from the Poisson distribution with a mean of 1. We then extracted SNV sites that were found in >5% of cancer cells sampled from different sectors of a tumor. Cancer cells were classified into the same clone if their genotypes were identical to each other. Clone frequencies in each tumor sector were obtained by counting the number of cells that comprise that clone.

By using these clone genotypes and clone frequencies, we generated mutant and wild-type read counts for each SNV that were input to clone prediction methods. We first computed expected SNV frequencies, in which we summed clone frequencies of clone genotypes with mutant bases at an SNV site and then divided it by two. Assuming that sequencing depth was 100, we generated total read count, by randomly drawing an observed total read count (*x*) from a Poisson distribution with a mean of the expected read count (100). We then sampled from a Binomial distribution with *x* trials and the expected SNV frequency to generate mutant read count. In total, we generated 90 TG datasets (30 datasets for each of linear, step, and constant model). True clone phylogenies were reconstructed by using maximum parsimony method in MEGA-CC^56^; all of these true phylogenies are shown in Supplementary Fig. S2.

#### Datasets with CNAs and LOH

We use G7 datasets and added copy number gains, losses, and LOHs to produce G7-CNA datasets. We used the same number of SNVs, clone phylogenies, and clone frequencies as the original G7 datasets. For each chromosome, we generated paternal and maternal chromosomes and selected 30 randomly sampled SNVs on either the paternal or the maternal chromosome. Sometimes a chromosome may contain fewer than 30 SNVs. We assigned copy number gains, copy number losses, and LOHs for each chromosome and also selected paternal or maternal chromosomes affected by a CNA event.

For each CNA event, the timing of occurrence was determined by randomly selecting a branch of the true clone phylogeny. The location of somatic mutations on sites and branches was also recorded on the true clone phylogeny. For each branch, the order of CNAs and somatic mutations were assigned randomly. Along this predefined history, we evolved the chromosomes and recorded the number of mutant and wild type copies at each SNV site. For example, at the time of a copy number gain event, we duplicated the affected chromosome. In the copy number loss case, the affected chromosome was deleted. For LOH, we deleted the affected chromosome and duplicated the other chromosome.

By using clone frequency (Fig. 1b) and copy number information of mutant and wild type bases at each SNV position, we calculated expected SNV frequencies sensitive to the CNAs and LOH. For example, at a position with one wild-type base copy and two mutant base copies, the multiplier to generate SNV frequency from the clone frequency would be 0.67 (2/3). This value was computed for each clone for a tumor sample with more than one clone, and the expected SNV frequency was computed as the summation of these values.

We also adjusted the expected total read counts for SNVs that were affected by CNAs. The expected total read count without CNAs was set to be 100. Since all of the clones may not have the same CNAs, we computed a weighted total read count for each clone by multiplying 100 with the clone frequency and the total copy number divided by two. We similarly computed a weighted total read count for normal cells, and we summed all of these values to generate an expected total read count for an SNV position.

By using these adjusted expected SNV frequencies and total read count, we generated the observed read counts. To introduce noise into the expected total read count, we randomly drew an observed total read count (*x*) from a Poisson distribution with a mean of the expected read count. We then sampled from a Binomial distribution with *x* trials and the expected SNV frequency to produce the final mutant read count.

### Selection of clone prediction methods and parameter settings

We selected clone prediction methods that have performed well in predicting clone genotypes from observed SNV frequencies or read counts of bulk sequencing data^35^. That is, we excluded methods that produce highly incorrect clone genotypes because such clone genotypes do not produce correct clone phylogenies. By this criterion, we excluded CITUP^57^, BayClone2^58^, Clomial^59^, Canopy^60^, cloneHD^61^, and AncesTree^54^ (see Supplementary Table S1 for the average number of SNV assignment errors per clone). We did not include methods that require prior information on the composition of SNV clusters (e.g., TrAp^62^) or those that require the use of another software to produce clone genotypes by ordering predicted clusters (e.g., PyClone^63^ and SciClone^64^). Lastly, we did not include methods that were designed for the analyses of single-cell sequencing data (e.g., SCITE^65^ and BEAM^66^), because clone deconvolution is not necessary for this type of data, while these methods focus on imputing missing data and minimizing SNV assignment errors in the inference of cell phylogenies^31,32^. These considerations resulted in the selection of seven clone prediction methods^13,34–39^. Each method was used with its default or recommended parameter settings. In MA datasets, we found many similar clone genotypes, so we used parameter settings that can differentiate similar clone genotypes. This modification was applied only for LICHeE and CloneFinder, as only these two methods include options for this purpose.

#### MACHINA

We used the PMH-TI mode in the MACHINA software, which infers clone genotypes from read count data^13^. The MACHINA software requires *a priori* identification of tumor sites as primary or metastatic for each sample. Since G7, G12, P10, and TG datasets were simulated without the consideration of primary and metastatic tumor evolution, we assumed that the primary tumor contained the root clone (e.g., clone A for G7 and G12 datasets) (Fig. 1a,c). When a root clone was not present in a dataset, we selected the clone that was most closely located to the root of a simulated phylogeny (P10 and TG datasets). If a root or closely related root clone was found within more than one tumor site, we randomly selected one of them to assign a primary tumor. For MA datasets, we provided the correct tumor site (primary or metastatic site, in which distinct metastatic tumor sites were accordingly distinguished). Note that MACHINA often produced a large number of solutions (>10 solutions per dataset) for G7, G12, MA, and TG datasets. In those cases, we first identified the best and worst solutions for each dataset, which were determined based on the average number of SNV assignment errors per clone. We reported the average error rate (see below) of the best and worst solutions.

For the analysis of TG datasets, we set the time limit for the search of solutions to be 10 seconds, because we found that 10 seconds were sufficient for MACHINA to find >1,000 candidate solutions (excluding the time to complete the whole analysis for each solution). Since the analysis of an extremely large number of solutions is not feasible in actual empirical data analysis, we considered that MACHINA was failed to produce a result when the number of solutions was >100. For datasets with <100 solutions, we further filtered into datasets that MACHINA completed the whole analysis of a single solution within 30 minutes because datasets that required >30 minutes per solution were rare among the other G7, G12, P10, and MA datasets. MACHINA did not complete the analysis for all of the TG-constant datasets, and we were able to obtain results only for five TG-step datasets. Therefore, we considered MACHINA failed for TG-constant and TG-step datasets. Next, we set three hours’ time limit to complete the analysis of all the solutions for a TG-linear dataset. This criterion removed only one linear dataset, which was considered as a failed dataset.

#### LICHeE

Following the default settings, we set the variant allele frequency (VAF) error margin the value 0.1^34^. SNVs were considered robustly present in a sample at VAF > 0.005 (robust SNVs), and the others were considered absent in a sample. SNVs with VAF > 0.6 were excluded. LICHeE groups SNVs based on the pattern of presence/absence of mutations across the samples and each SNV group was required to contain at least two robust SNVs. LICHeE also clusters SNVs by VAF similarities. We required that an SNV cluster contained at least two SNVs unless an SNV was sample-specific. All the SNV groups/clusters were initially kept in the network. Two groups/clusters could collapse when mean VAF difference was <0.2.

LICHeE did not produce clonal compositions of samples (i.e., clone frequencies). Thus, we estimated clone frequencies using the relationship ½*f* × M = *V*, where *f* is a two-dimensional matrix of estimated clone frequencies of the samples, M is a matrix of predicted clone genotypes, and *V* is the observed SNV frequency^33^. The equation above applies to cases where the variants are free of copy number alterations (CNAs)^33^, which is the case for our datasets. We estimated *f* through the regression of *V* to a function of M and *f* ^67^. Clone frequencies were estimated excluding SNVs with small total read count (<50) and mutant read count (<2), because those observed SNV frequencies were not reliable. When ancestral clones were predicted to co-exist with their descendant clones within a sample, we tested if these ancestral clones were spurious. Between a pair of ancestral and descendant clones, we compared observed SNV frequencies that are unique to the descendant clone and those shared with the ancestral clone. We used the expectation of higher observed SNV frequencies on shared (mutations that were found in both clones) than on unique mutations (mutations that were found in only a descendant clone; *t*-test) to discover the spurious presence of ancestral clones. When the differences between SNV frequencies were not significant (*P* > 0.05), the ancestral clones were removed. Also, we discarded clones present at low frequencies (<2%).

In the analyses of MA datasets, only SNVs with zero SNV frequency were considered to be robustly absent from a sample, and SNVs with >0.0001 frequency were considered to be robustly present in a sample (robust SNVs). All SNVs were examined regardless of their observed frequency. The minimum number of SNVs per cluster/group was set to one. Two SNV clusters were collapsed when mean SNV frequency differences were less than 1%. We did not discard any ancestral clones.

In the analysis of TG datasets, LICHeE sometimes produced clone phylogenies that did not contain any sequential or parallel mutations. These inferred clone phylogenies were completely a star-shape or line-shape, which was incorrect. Since the error rate of ordering sequential or parallel mutations became infinite for these phylogenies, we considered that LICHeE failed on these datasets.

#### CloneFinder

We estimated clone genotypes using SNVs with at least 50 reference read counts and two mutant read counts, and we discarded clones when estimated clone frequencies were <2%^35^. To analyze MA datasets, we did not combine similar clone genotypes or discard clones. We used all reads.

#### Treeomics

We used the option of enabling subclone detection^36^.

#### PhyloWGS

The fraction of expected reference allele sampling from the reference population and the variant population were 0.999 and 0.4999, respectively^37^. We set a copy number equal to one (heterozygous mutant allele). As PhyloWGS did not produce clone frequencies, we computed clone frequencies using the approach described for LICHeE (see above). Since PhyloWGS produced five solutions for each dataset, we reported an average score over these five solutions.

#### Mixed perfect phylogeny (MixPhy)

We performed analyses in MixPhy (v0.1) with the option of a heuristic algorithm. As the input file requires a binary matrix of tumor sample genotypes (presence/absence of mutation), we provided correct sample genotypes, assuming that there were no false positive or false negative detections of mutations^38^.

#### Cloe

We used the option to perform mutation clustering to improve runtime^39^. We applied Cloe with 100,000 iterations and used the default four parallel tempered chains. For G7 datasets, we used 10,000 iterations, because it converged without performing 100,000 iterations. For the posterior evaluation of MCMC sampled trees, the burn-in of MCMC chains was 0.5, and chain thinning was 20. The maximum number of clones for a dataset was set to the true clone count. MA datasets were not analyzed, because Cloe did not converge for a few large datasets (17 and 18 clones per dataset), even when we used 400,000 iterations. Similarly, Cloe did not converge for a G7 dataset with CNAs, even when we tried up to 5,000,000 iterations, which required a few days of computation.

### Inference of consensus clone phylogenies

We used GraPhyC with the options of “path”, “parent-child”, “ancestor-descendant”, and “clonal”^50^. We used MA datasets and computed consensus trees of the best-performing methods (CloneFinder, MACHINA, Treeomics, and LICHeE; smaller overall MLTED scores than the other methods (Fig. 3)). Since MACHINA often produced more than one solution, we first computed consensus trees of these solutions.

### Evaluation of predicted clone phylogenies

We compared each predicted clone phylogeny with the respective true clone phylogeny by using the following four metrics.

#### Multi-labeled tree edit distance (MLTED)

A clone phylogeny is often viewed as a mutational tree^44^ in which all the mutations are mapped along branches. Mutational trees are useful when the number of tips in the inferred clone phylogeny differs from the true phylogeny and when the sequences of the inferred clones do not match all the true clones. We used the Multi-labeled Tree Edit Distance (MLTED score) for comparing the inferred and the true tree, as it has been designed to evaluate clone trees^68^, available at https://github.com/khaled-rahman/MLTED. This algorithm requires that the inferred tree contains the same set of mutations as in the true tree. Because of errors in clone sequence predictions, some mutations were not assigned to any branch in the inferred tree. These mutations were placed at the root of the inferred mutational tree.

#### The error rate of ordering mutations

We generated all possible pairs of SNVs (mutations) and classified them into three possible types, i.e., concurrent, sequential, and parallel (see Fig. 2 for some examples). Concurrent mutations are those that occurred on the same branch (irrespective of their order), whereas sequential and parallel mutations are those that occurred on different branches of the clone phylogeny. More specifically, two mutations are sequential if one occurred on the ancestral branch and the other on its descendant branch, but multiple intervening branches may separate them. Two mutations are parallel if they are found on sibling lineages that have descended from their most recent common ancestor. Any true mutation pair not found in the inferred tree was classified as “unassigned.”

We estimate the error rate of ordering concurrent, sequential, and parallel mutations, separately. In each category, we first scored the number of true mutation pairs that were not present in the inferred tree and divided it by the total number of true mutation pairs. Then, we scored the number of mutation pairs that were incorrect and divided it by the total number of inferred mutation pairs. Then, the average of these two proportions was used as the error rate of ordering the given type of mutations. Similar measures have been used to evaluate clone prediction methods in previous studies^34^.

#### Advanced Tree vector (TreeVec)

We also evaluated the accuracy of branching patterns (topology) in inferred clone phylogenies (clonal lineage trees^44^). For this purpose, we first mapped inferred clone genotypes to the true clone genotypes, because inferred clone genotypes never perfectly match the true clone genotypes. We mapped each inferred clone genotype to its most similar true clone genotype in a two-step process^69^. First, each true clone genotype was compared to all the inferred clone genotypes, and the two clones with the smallest difference were paired. When the number of inferred clones was greater than the number of true clones, the remaining inferred clones were paired with the most similar true clone genotype. For uniformity, we reconstructed inferred clone phylogenies by using predicted clone genotypes produced by each method. Because mutations arose only once in the computer simulated data, the maximum parsimony analysis was suitable^70^ and was performed using MEGA-CC^71^. All the clone phylogenies were rooted using germline sequences (normal cells) as outgroups. In inferred clone phylogenies, we labeled tips with clone annotations. When an inferred clone genotype had two different annotations, we duplicated the genotype in an inferred clone phylogeny, i.e., the corresponding tip was duplicated. Also, two inferred clone genotypes might have the same annotation. In this case, two tips in an inferred clone phylogeny were labeled identically.

Among various tree distance computation methods for phylogenies^72^, we selected the advanced TreeVec distance developed by Kendall *et al*.^69^, because TreeVec allowed more than one tip with identical labels. Briefly, TreeVec distance computation first collapsed any monophyletic clade(s), i.e., a clade with tips that had an identical label. Then, the traditional TreeVec distance^73^ was computed, which counted the number of branches (edges) between the root and the node of the most recent common ancestor (MRCA) of a pair of clones. For all pairs of clones, the Euclidean metric between inferred and true counts was computed. We used the treespace software^74^ to compute this advanced TreeVec distance.

#### Robinson and Foulds (RF) distance

We also computed RF tree distance, because it is widely applied in the evaluation of species phylogenies^75^. We used PhyloNET software^76^ to count the number of partitions that were common and different between the true and the inferred phylogeny. The RF distance is the number of differing partitions divided by the total number of partitions in the two phylogenies. Note that RF distance computation requires that both the inferred and the true clone phylogenies contain the same number of tips (clones). However, inferred clone phylogenies may contain more tips than the respective true phylogenies, when more than one tip is assigned an identical clone annotation (i.e., more than one inferred clone genotype was similar to a true genotype). When there were too many tips in the inferred tree, we retained only those tips that showed the highest similarity to the true clone genotypes, such that each true clone genotype was matched with exactly one inferred genotype.

### Empirical data analyses

We obtained an empirical dataset (patient A7 dataset^30^; https://github.com/raphael-group/machina), which contained 478 copy-neutral SNVs. This dataset contained SNV frequencies of one primary tumor sample (breast) and four metastatic tumors (lung, liver, rib, and brain), for which clone phylogenies and clonal composition of each sample were previously reported^30^. For real data, true clone genotypes were not available, so we annotated each clone on the inferred phylogeny based on the sample(s) that contained it (Fig. 12a) in order to compare the reported phylogeny^30^ with those inferred by the clone prediction methods listed in Table 1.

Observed read count information of two empirical datasets (patient 3 and patient 9 datasets) was obtained from the Supplementary Information of ref. ^51^. Patient 3 contained eleven tumor samples from adnexa site, cul de sac site, left fallopian tube fimbriae site, left ovary site, two omentum sites, right fallopian tube site, three right ovary sites, and sigmoid colon deposit site, where the left ovary site was reported as the primary tumor site in the original study^51^. Patient 9 contained five tumor samples from two left ovary sites, two omentum sites, and right ovary site, where the left ovary site was reported as the primary tumor site^51^. The numbers of SNVs were 131 and 183 SNVs, for patient 3 and patient 9, respectively. The original study^51^ inferred clone phylogenies and confirmed them by using single-cell sequencing data. We annotated each clone on the inferred phylogeny based on the sample(s) that contained it (Fig. 12j,n).

## Supplementary information

Supplementary information.

## Data Availability

The G7, G12, P10, MA, TG, G7-CNA, and MA-50x datasets and a pipeline to replicate our study (ClonePhyTester) are available on the website of the ClonePhyTester software (https://github.com/SayakaMiura/ClonePhyTester).
