# Supplementary material for: Power and pitfalls of computational methods for inferring clone phylogenies and mutation orders from bulk sequencing data
Source: Sci Rep. 2020 Feb 26;10:3498. doi: 10.1038/s41598-020-59006-2 (PMC7044161; doi:10.1038/s41598-020-59006-2)
Supplement: Supplementary file 1 — Supplementary information. [file 41598_2020_59006_MOESM1_ESM.pdf]

Supplementary Information

Power and pitfalls of computational methods for inferring clone phylogenies and mutation orders from bulk sequencing data

Sayaka Miura[1,2], Tracy Vu[1,2], Jiamin Deng[1,2], Tiffany Buturla[1,2], Olumide Oladeinde[1,2], Jiyeong Choi[1,2], and Sudhir Kumar[1,2,3*]

[1]Institute for Genomics and Evolutionary Medicine, [2]Department of Biology, Temple University, Philadelphia, PA 19122, USA.[3]Center for Excellence in Genome Medicine and Research, King Abdulaziz University, Saudi Arabia.

Supplementary Table S1: Average number of SNV assignment error per clone

	Dataset			
Method	G12	G7	P10	MA
CloneFinder	0%	2%	3%	4%
MACHINA	2%	4%	3%	3%
TreeOmics	1%	6%	4%	5%
LICHeE	2%	10%	3%	4%
MixPhy	11%	8%	6%	6%
PhyloWGS	9%	6%	5%	5%
Cloe	7%	1%	4%	-
CITUP	11%	5%	7%	-
BayClone2	8%	8%	7%	-
Clomial	-	9%	8%	-
Canopy	15%	9%	-	-
cloneHD	16%	13%	13%	-
AncesTree	17%	25%	20%	-

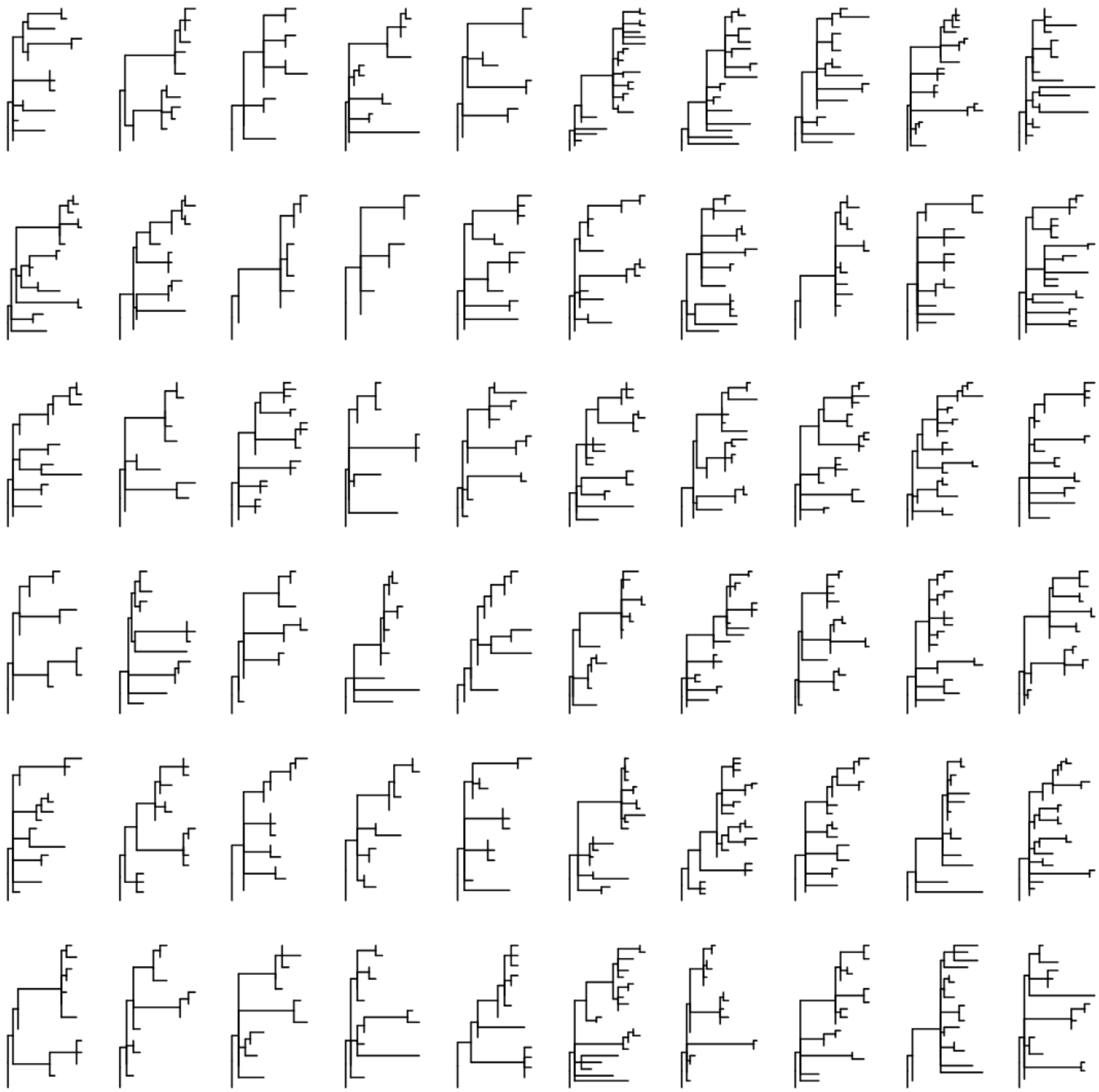

Figure S1: All clone phylogenies in MA datasets.

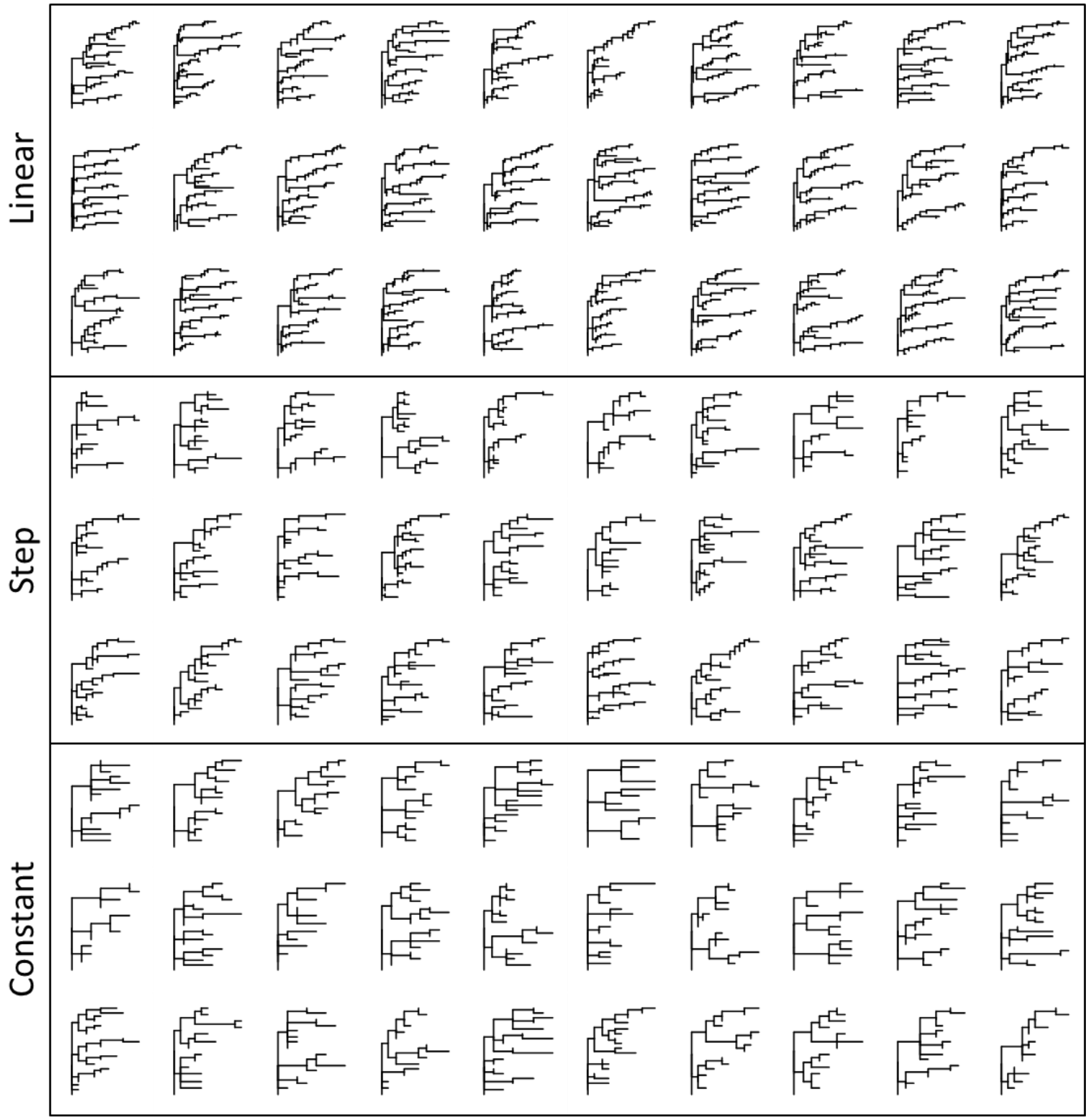

Figure S2: All clone phylogenies in TG datasets.

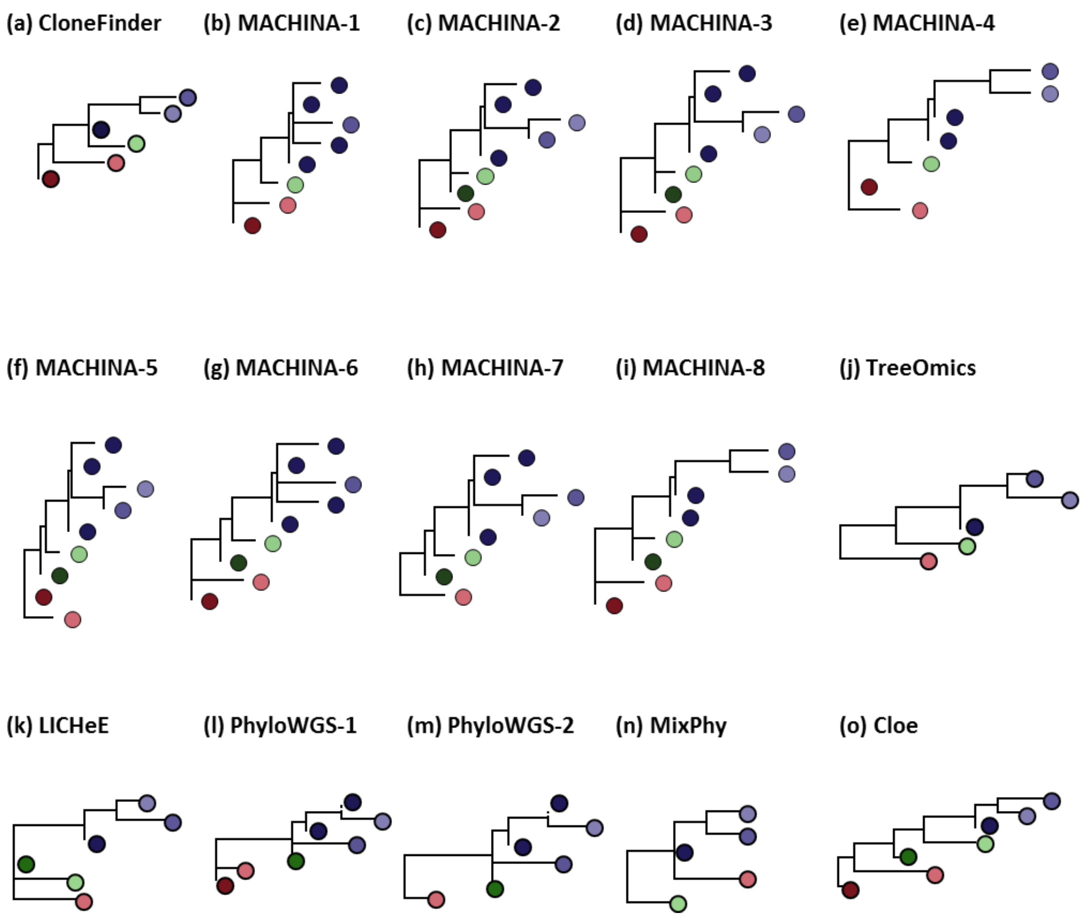

Figure S3: Predicted clone phylogenies on G7 datasets. The average proportion of SNV assignment errors per clone was computed for each dataset. For each method, we selected a dataset that showed the most similar SNV assignment error to that of the average across the G7 datasets. For MACHINA, the selected dataset produced eight different solutions, and we showed all of them (**b-i**). See **Figure 1** for the color code for each clone and correct topology of the phylogeny.

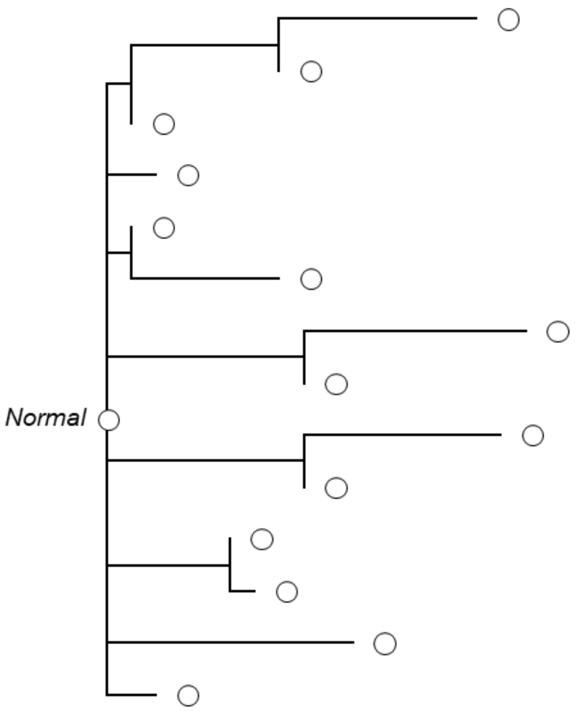

Figure S4: An inferred phylogeny of MixPhy for a G12 dataset.

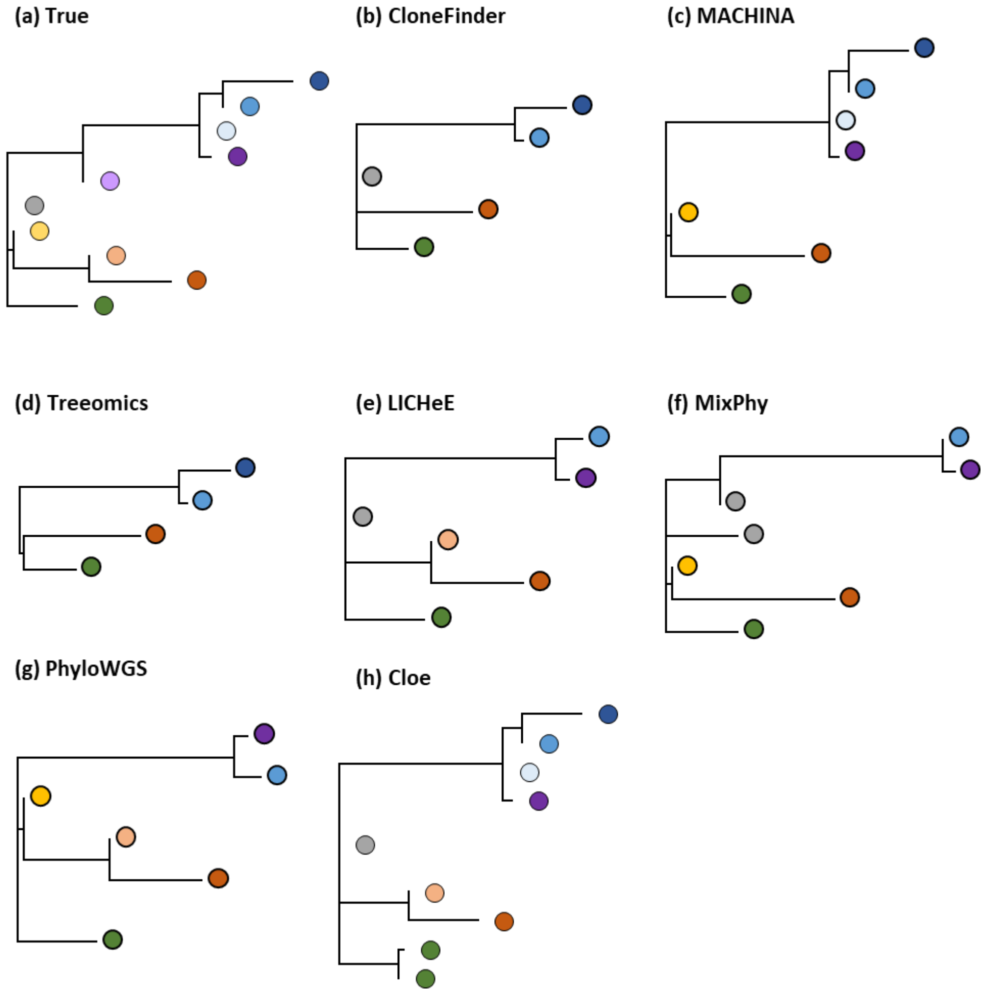

Figure S5: True and predicted clone phylogenies of a P10 dataset. This dataset contained six ancestral clones (**a**), and many of these ancestral clones were not identified by these clone prediction methods (**b-h**).

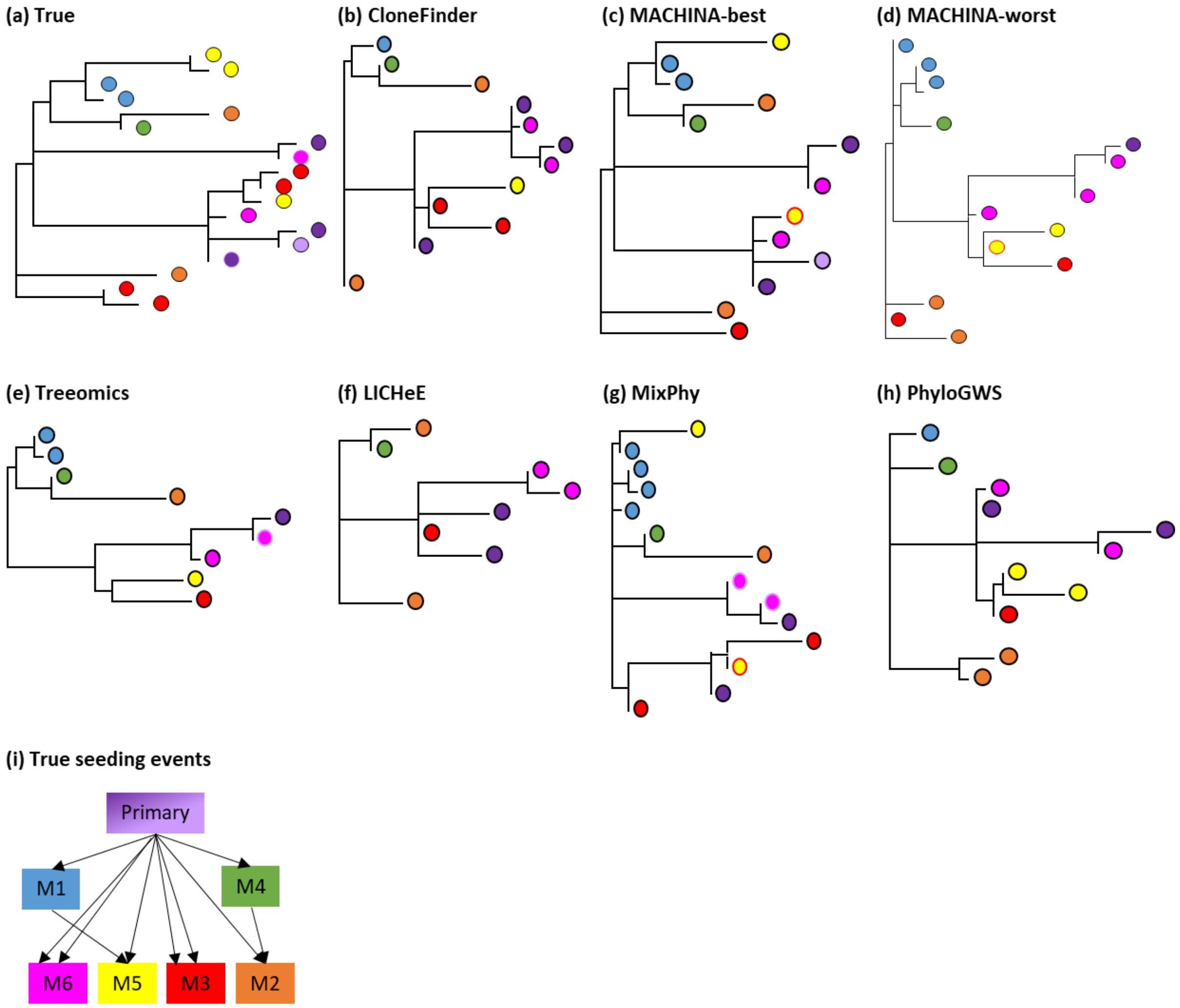

Figure S6: True and predicted clone phylogenies of a MA dataset. This dataset contained four metastatic tumors that arose with polyclonal seeding events (**i**). In total, there were six metastatic tumors (M1-M6; one section per tumor) and two sections from the primary tumor. In the true clone phylogeny, clones were colored based on the tumor sites that contained them (**a**). For the predicted clone phylogenies, we annotated each clone (**b-h**). Colors were corresponded to those in the true phylogeny. MACHINA produced 870 solutions for this dataset and we selected the best and worst solutions based on the number of SNV assignment errors per clone.

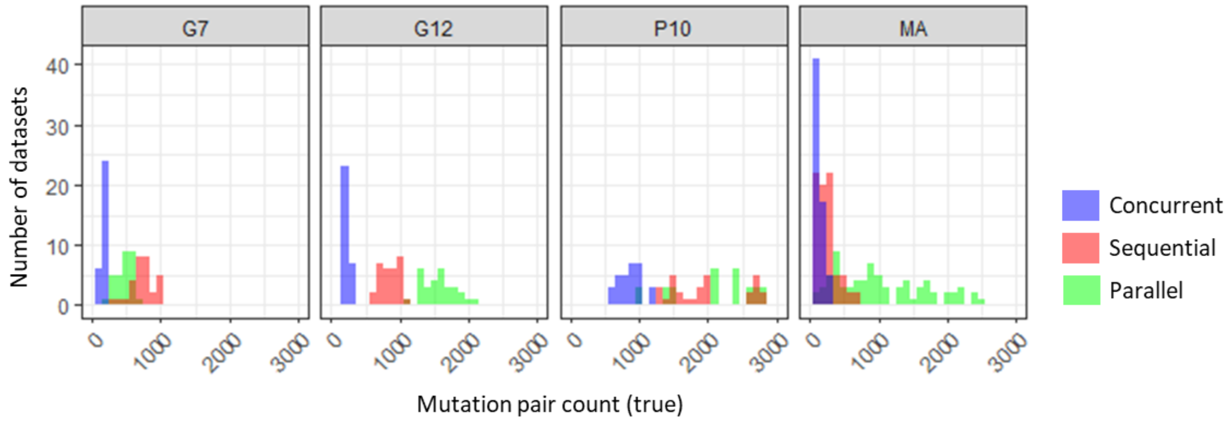

Figure S7: The numbers of true concurrent, sequential, and parallel mutation pairs within a dataset.

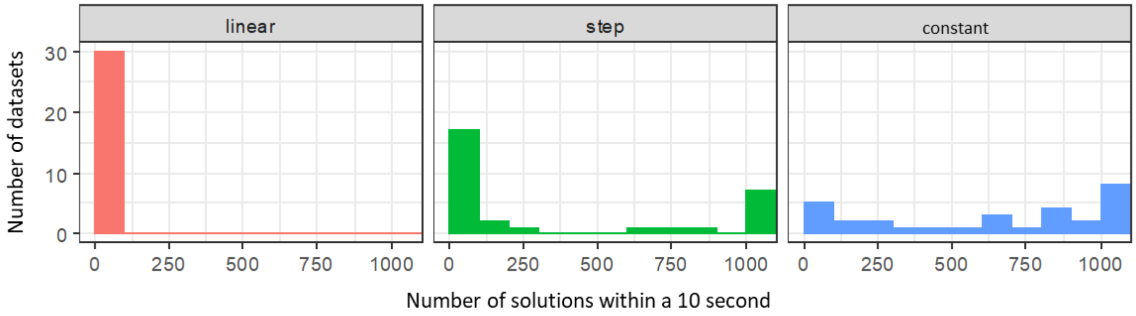

Figure S8: The numbers of solutions that MACHINA produced within a 10 second. Linear, step, and constant datasets from TG datasets were used.
